# Selective reduction of APP-BACE1 activity improves memory via NMDA-NR2B receptor-mediated mechanisms in aged PDAPP mice

**DOI:** 10.1016/j.neurobiolaging.2018.11.011

**Published:** 2019-03

**Authors:** Charles E. Evans, Rhian S. Thomas, Thomas J. Freeman, Martha Hvoslef-Eide, Mark A. Good, Emma J. Kidd

**Affiliations:** a School of Psychology Cardiff University, Cardiff, UK; b School of Pharmacy & Pharmaceutical Sciences, Cardiff University, Cardiff, UK; c Department of Applied Sciences, University of the West of England, Bristol, UK; d Department of Biosciences, University of Oslo, Olso, Norway

**Keywords:** Amyloid precursor protein, BACE1, βCTF antibody, Amyloid, Hippocampus, NMDA, Memory

## Abstract

β-Amyloid (Aβ) accumulation is an early event of Alzheimer’s disease (AD) pathogenesis. Inhibition of Aβ production by β-secretase (BACE) has been proposed as a potential therapeutic strategy for AD. However, BACE inhibitors lack specificity and have had limited clinical benefit. To better study the consequences of reducing BACE metabolism, specifically of APP, we used an antibody, 2B3, that binds to APP at the BACE cleavage site, inhibiting Aβ production. 2B3 was administered either directly into the lateral ventricles or by intraperitoneal injection to (platelet-derived growth factor promoter hAPP717V (PDAPP) mice and WT mice. 2B3 reduced soluble Aβ40 and βCTF (β-amyloid derived C-terminal fragment) and improved memory for object-in-place associations and working memory in a foraging task in PDAPP mice. 2B3 also normalized the phosphorylation of the N-methyl-D-aspartate receptor NR2B subunit and subsequent extracellular signal–regulated kinase signaling. The importance of this NR2B pathway for OiP memory was confirmed by administering the NR2B antagonist, Ro25-6981, to 18-month-old WT. In contrast, 2B3 impaired associative recognition memory in young WT mice. These data provide novel insights into the mechanism by which selective modulation of APP metabolism by BACE influences synaptic and cognitive processes in both normal mice and aged APP transgenic mice.

## Highlights

• Inhibition of APP metabolism by ICV 2B3 reduced Aβ and βCTF production
• 2B3 prevented the onset of memory impairments in PDAPP mice
• 2B3 restored NR2B receptor phosphorylation and subsequent ERK activity
• NMDA-NR2B receptor mechanisms contributed to object-in-place memory
• Directly inhibiting APP-BACE metabolism provides a therapeutic strategy for AD

## 1 Introduction

The excess accumulation of β-amyloid (Aβ) in the brain with age is considered an important factor in the cascade of cellular, neural network, and cognitive changes that characterize the early stages of Alzheimer’s disease (AD) (Hardy and Selkoe, 2002, Selkoe, 2001). Aβ is produced by the proteolytic cleavage of amyloid precursor protein (APP) by the β-secretase cleavage enzyme (BACE1). BACE1 is located in the presynaptic terminals of neurons and is thought to be critical for the production of Aβ and subsequent disruption of synaptic connectivity that characterizes early-stage AD (Sadleir et al., 2016).

One approach to altering APP metabolism is through the use of BACE1 inhibitors (Yan and Vassar, 2014). However, this approach has been problematic because BACE1 has multiple substrates other than APP (Hemming et al., 2009). Although BACE1 modulation in people with clinically diagnosed dementia appears to lack therapeutic efficacy, the targeting of this pathway in the elderly with cognitive decline or those at high risk of dementia remains an area of current interest. Therefore, understanding the effects of selectively modifying BACE1-APP processing on synaptic function and cognition is vital to understanding the mechanism and the conditions under which such an intervention may have therapeutic value.

In the present study, we used a monoclonal antibody, 2B3, directed against the BACE1 cleavage site of APP to reduce BACE1 cleavage without influencing other BACE1 substrates (Thomas et al., 2013, Thomas et al., 2011b). We hypothesized that in vivo administration of 2B3 either directly into the brain or systemically into an aged mouse model of excess Aβ accumulation would (1) reduce Aβ production, (2) improve aberrant synaptic processes in the hippocampus, and (3) improve visuospatial associative recognition memory. In experiment 1, we first established the age of onset of a selective associative (object-in-place [OiP]) recognition memory deficit in transgenic platelet-derived growth factor promoter hAPP717V (PDAPP) mice; a task that relies on a brain network that includes the hippocampus (Barker and Warburton, 2011). In experiment 2a, we used the mice from experiment 1 to assess the effects of intracerebroventricular (ICV) administration of the antibody, 2B3, on associative memory dysfunction in aged PDAPP mice. In experiment 2b, we addressed the question of whether disruption of endogenous APP processing by 2B3 in normal mice influenced associative recognition memory. In experiment 3, we investigated the effects of chronic intraperitoneal (IP) administration of 2B3, initiated before the onset of behavioral impairment, on the subsequent development of cognitive dysfunction in aged PDAPP mice. Finally, experiment 4 used a within-subject Latin-square design to test the hypothesis that disruption of glutamate signaling through N-methyl-D-aspartate (NMDA) NR2B receptors, evident in PDAPP mice from experiments 2 and 3, was required for normal associative recognition memory in WT mice. Our results show for the first time that selective reduction in APP metabolism by BACE1 using steric hindrance both improved and protected mice from memory dysfunction and altered synaptic NMDA-NR2B expression in PDAPP mice.

## 2 Materials and methods

### 2.1 Animals

Male PDAPP mice (Games et al., 1995), and their WT littermates, were bred and maintained on a C57Bl/6 genetic background as previously described (Evans et al., 2018). Mice were housed either individually (if they showed signs of aggressive behavior) or in groups. One WT and one transgenic mouse were housed separately. All animals were housed using standard environmental and cage conditions, including nesting cardboard tubes and clean bedding. Behavioral testing was carried out during the light hours of the cycle. Animals were maintained according to the UK Home Office under the Animal Scientific Procedures Act (1986) and EU regulations.

Before assignment to groups, the mice were genotyped according to protocols described previously (Evans et al., 2018). Briefly, an ear biopsy sample was collected from each mouse at 6-8 weeks of age as part of animal husbandry identification procedures, which was then digested and DNA extracted using DNeasy Blood and Tissue kits (Qiagen). A polymerase chain reaction was used to amplify the human *APP V717F* transgene DNA. PDAPP-specific primers forward: 5’-ATCTGGCCCTGGGGAAAAAAG-3’ and reverse: 5’-GATGTCCTTCCTCCTCTGTTC-3’ (Eurofins, Wolverhampton, UK) amplified the *hAPP V717F* mutation. Control primers for *MusA-Actin* forward: 5’-CACCACACCTTCTACAATGAGCTG-3’ and reverse: 5’-TCATCAGGTAGTCAGTGAGGTCGC-3’ (Eurofins) targeted *MusA-Actin.*

Experiment 1 established the age of onset of cognitive impairment, and experiment 2 assessed the effects of ICV infusion of 2B3 on cognition in the same animals. ICV administration was considered the most appropriate means to ensure the delivery of the antibody in the brain and thus provide a test of its putative actions in vivo as evidenced from our previous cell culture experiments. Experiments 1 and 2a consisted of 2 replications: The first replication comprised PDAPP (n = 14) and WT littermates (n = 15), which underwent a battery of object memory tests at 6-8, 10-12, and 14-16 months of age. Subsequently, these mice received ICV 2B3 (Thomas et al., 2011b, Thomas et al., 2013) or vehicle at 17-18 months of age using osmotic minipumps (experiment 2a). A second replication of 17- to 18-month-old WT (n = 8) and PDAPP (n = 8) mice received the same schedule of prior training (age-of-onset testing; data not shown) and 2B3 administration. Together, these subgroups yielded a final total number of 11 WT untreated, 10 WT vehicle, 11 vehicle PDAPP vehicle, and 10 2B3 PDAPP mice (1 WT and 1 PDAPP mouse died during osmotic minipump implantation). A separate third cohort of PDAPP mice was used only to confirm and quantify aging-related changes in human Aβ levels at 3 (n = 5), 7 (n = 7), 11 (n = 7), and 15 (n = 7) months of age; these mice did not undergo behavioral testing. For experiment 2b, a separate cohort of 5- to 6-month-old WT mice were administered either 2B3 or a control mouse monoclonal IgG1κ antibody (Millipore; MAB201) by osmotic minipump to investigate the effects on cognition of disrupting endogenous mouse APP processing with 2B3. Experiment 2b consisted of 8 WT mice that received the control IgG antibody and 8 mice that received ICV 2B3 via osmotic minipumps. As 1 mouse died postoperatively before the start of behavioral testing, it was removed from subsequent analyses. The final *n*'s were, therefore, 8 WT control and 7 WT 2B3.

**Figure 1:**
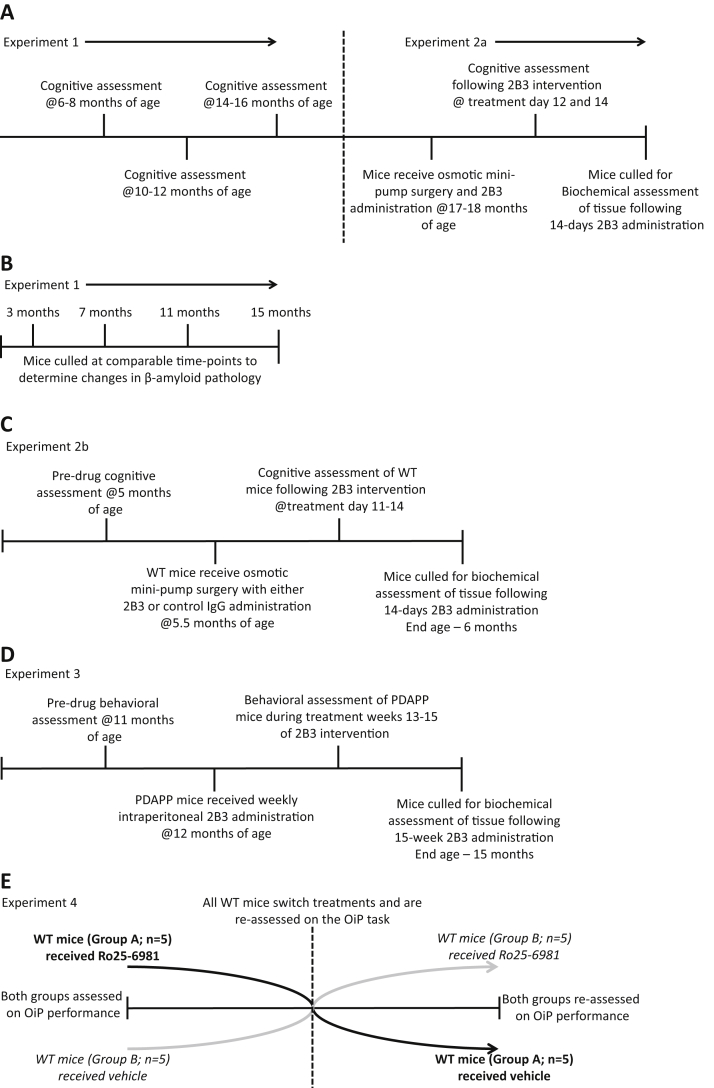
Schematic illustration detailing experimental designs used in this study. (A) Experiment 1 assessed cognitive performance (object-novelty and OiP performance) of PDAPP and WT littermate controls at 3 age points across the study. Experiment 2a used the same mice to assess whether 2B3 (icv) infusion by osmotic minipumps could improve OiP memory and pathology in PDAPP mice. (B) A separate cohort of PDAPP mice was used to exclusively determine the age-related changes of β-amyloid pathology in the hippocampus of PDAPP mice using time points comparable to the behavioral time points used in seperate experiments. (C) A separate cohort of WT mice was administered 2B3 to assess its effect on normal endogenous mouse APP processing and memory. These mice first received behavioral testing (object-novelty and OiP memory) prior to implantation of osmotic minipumps. Subsequently, they were administered 2B3 or IgG control antibody for 14 days. Memory function was then reassessed before culling and assessment of endogenous APP metabolism. (D) The effects of 2B3 administered longitudinally via a peripheral (i.p.) route on cognition was assessed in a separate cohort of PDAPP mice. These mice first received pretreatment behavioural testing (object novelty, OiP, and foraging behavior tasks) at 11 months of age. After which, the mice were administered 2B3 or vehicle i.p. for 15 weeks. During treatment weeks 13-15, memory function was reassessed, following which, the mice were culled and brain pathology was analysed. (E) Experiment 4 assessed the role of the NR2B receptor in OiP memory in normal WT mice. This was carried out in a Latin-square design whereby the cohort was split into 2 groups (A and B; each group n = 5). Group A were initially assessed on the task after Ro25-6981 administration, while group B after vehicle treatment. After this initial assessment, Ro25-6981 was then administered to group B mice, while group A mice now received vehicle. Abbreviations: OiP, object-in-place; WT, wild type.

In experiment 3, PDAPP (n = 19) and WT littermate controls (n = 20) were used to assess the protective effects of long-term administration of 2B3 that was initiated before the onset of cognitive impairment. As long-term administration was not feasible using an ICV minipump delivery method, 2B3 was administered to PDAPP mice (n = 9) by once weekly IP injection at 20 mg/kg for a period of 15 weeks from 11 months of age. Vehicle was administered at an equal volume in control WT (n = 10) and PDAPP mice (n = 10). An additional WT untreated group (n = 10) was included to assess the potential impact of IP treatment on performance. The initial behavioral tests were carried out at 11 months of age, before 2B3 administration. All treatment groups were then subsequently matched in terms of their object contact times and discrimination ratios (data not shown). After a further 15 weeks of IP injections, memory function was tested at 15 months of age. These mice were tested on object memory tasks and a spatial working memory foraging task (Evans et al., 2018) to establish the generality of the behavioral effects of 2B3 administration across different performance measures. A breakdown of the experimental designs is illustrated in Figure 1.

Experiment 4 evaluated the hypothesis that NMDA-NR2B receptor function was necessary for associative recognition memory in WT mice. A naïve cohort of 18-month-old WT littermate controls (n = 10) was administered the NR2B receptor antagonist Ro25-6981 or vehicle 30 minutes before behavioral testing. The experiment was run as a within-subject Latin-square design. A Latin-square is a commonly used experimental design to assess the effects of drug(s) and vehicle in the same animals on performance in a counterbalanced fashion.

Mice were given a 48-hour rest interval between each injection as a washout period before the next phase of testing. Novel objects were used in each phase of drug testing, and their order was counterbalanced across animals.

### 2.2 Behavior

**Figure 2:**
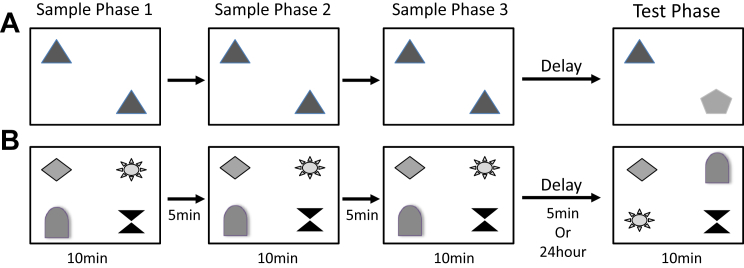
Schematic diagram illustrating object novelty and object-in-place recognition memory tasks. To assess novel object memory (A) mice were presented with 2 identical objects for 3 separate 10-minute sample phases, each separated by a 5-minute delay during which the mouse was returned to its home cage. Five minutes or 24 hours later, novel object memory was tested by presenting the mouse with 1 familiar object and one novel object and recording the contact time with each. Object-in-place memory (B) was tested in a similar fashion except 4 unique objects were used. In the test phase, 2 objects were switched spatial location and 2 remained in the same familiar locations. The contact times with objects in novel and familiar locations were recorded.

In experiments 1 and 2, mice received 2 different object-based memory tasks; a novel object recognition task and a visuospatial OiP task (Barker and Warburton, 2011, Good and Hale, 2007, Hale and Good, 2005) (Fig. 2A and B). Briefly, before testing object memory, animals were habituated to the arena (60 cm × 60 cm square × 40 cm high). Mice were allowed to explore the arena freely for 10 minutes on day 1. Mice were then habituated for 2 consecutive days to the arena containing 2 identical objects for 10 minutes each day. Each mouse received 2 rounds of testing on each task, 1 day with a 5-minute and 1 day with a 24-hour delay period (in a counterbalanced order). The objects were a collection of every day items and ornaments as described previously (Hale & Good, 2005).

#### 2.2.1 Novel object recognition

To assess object novelty versus familiarity discriminations, 2 identical objects were placed in the center of the arena and mice were allowed to explore the objects for 10 minutes during the sample phase. Mice were given a total of 3 sample phases, each separated by a 5-minute retention interval, during which the mouse was returned to its home cage. Following the third sample phase, mice received either a 5-minute or 24 hour delay period, the order of which was counterbalanced within groups. During the test phase, 1 familiar and 1 novel object were then placed into the arena in identical spatial locations as the sample objects (Fig. 2A). The mouse was then returned to the arena for 10 minutes to explore the object array.

#### 2.2.2 Associative OiP memory

Four different objects were placed in the center of the arena in a square formation. Each object was approximately 15 cm from the walls and 25 cm apart from each other. Mice were placed in the center of the arena and exposed to the 4 different objects for 3 sample phases and a test phase as described for the object novelty test. In the test phase, conducted 5 minutes after the sample phase, the spatial location of 2 objects that were positioned diagonally opposite each other was switched (Fig. 2B). The specific pair of objects that underwent the switch was counterbalanced within and between groups.

#### 2.2.3 Behavioural measures

For both recognition tasks, the animals' exploratory behavior was assessed during both the sample and test phases. Time spent exploring the objects was recorded in each phase. Object exploration was defined according to the methods described previously by Ennaceur and Delacour (1988). In brief, object contact was defined as when an animal was within a 2 cm radius of the object and directly facing, sniffing, gnawing, but not climbing or sitting on, the objects. A discrimination ratio (DR) was used to index the animals' discriminative performance in the test phase that was independent of individual differences in object contact times; this was calculated as the time spent exploring the novel object (or objects in novel locations)/the time spent exploring all objects. All objects were cleaned before each phase of testing to reduce the use of odor cues introduced from handling the objects.

#### 2.2.4 Foraging behavior

To establish the generality of the cognitive changes promoted by 2B3 administration, mice that received longitudinal IP 2B3 injections were also assessed on a foraging-based spatial working memory task sensitive to age-dependent changes in PDAPP mice (Evans et al., 2018). Briefly, throughout the training and test phase, mice were water-deprived to approximately 90% of their pre-training weight. Water was given for 4 hours immediately after training or testing each day. Mice were trained to forage from white ceramic pots (6.5 cm diameter, 3.5 cm depth; Lakeland, UK), which were mounted on a wooden cube base. Pots were secured to the floor of the cage/arena with blue-tack. In the initial training stages (day 1-3), pots containing 10 mL of water were placed in the home cage of mice for 1 hour to encourage mice to consume liquid rewards from within the pot. The following 3 days (day 4-6), 2 pots were baited with a 30 μL liquid reward (1:3 sweetened condensed milk [Nestle] solution, prepared in water; H_2_0) in an open arena with 1-cm-deep sawdust covering the arena floor. Mice were placed in the center of the arena and given 3 minutes to forage from each pot. Mice were removed once the liquid reward was consumed from each pot or the 3-minute limit was reached. Mice were given 1 training session/day. On each day, the location of the pots was moved to a new location to prevent the development of any systematic search bias in the test phase.

Mice were then tested over the next 4 consecutive days with 1 session per day. During these sessions, the arena was set up with 6 pots arranged in a circular pattern, each 20 cm apart. The mouse was placed in the center of the arena and allowed to forage pots until they had consumed all 6 rewards or until a 10-minute period had elapsed from when the first pot was foraged. Following the trial, mice were returned to their home cage. The pots were then wiped clean with 70% ethanol wipes, and the milk solution replenished before the next mouse was tested. All test sessions were recorded onto a DVD player using an overhead camera.

Foraging behavior was defined as a mouse jumping onto the rim of a pot and directing its nose in toward the bottom to consume a reward. An error was defined as a mouse returning to forage in a pot that had previously been foraged. A full description of errors can be found in Evans (2018).

### 2.3 Antibody production and administration

Full details of the immunization protocol, hybridoma development, antibody characterization, production, and purification are provided elsewhere (Thomas et al., 2006, Thomas et al., 2011b, Thomas et al., 2013).

In Experiments 2a and 2b, purified and sterilized 2B3 (1.2 mg/mL), control monoclonal IgG1κ antibody (experiment 2b) and vehicle (experiment 2a) were administered intracerebroventricularly via a 28G cannula surgically implanted into the left lateral ventricle (Alzet; 0004760). The minipumps were attached to the infusion cannula via a catheter and implanted subcutaneously between the scapulae. Minipumps were filled with 200 μL of antibody or vehicle (Model number 1002; Alzet, California, USA; flow rate 0.25 μL/hour for a period of 14 days). In experiment 2a, the vehicle was sterile PBS and was administered to both PDAPP mice (n = 11) and WT littermate control groups (n = 10). In experiment 2b, the WT control group received ICV infusion of a monoclonal IgG1κ.

In experiment 3, mice received a weekly IP injection of 2B3 for 15 weeks. 2B3 was produced by ascites (ProMab, California, USA), before being purified and sterilized as described previously. PDAPP mice were administered 2B3 (IP) at a dose of 20 mg/kg at an average concentration of 3 mg/mL. Sterile PBS was administered at the same volume to vehicle PDAPP and WT mice control groups. After the completion of behavioral testing and 3 days after the final IP injection of 2B3, brain tissue was collected from all mice for biochemical analyses.

### 2.4 Surgical procedure

During stereotaxic surgery, the mouse was anesthetized using an isoflurane/O_2_ mix, and a small hole was drilled through the skull of the animal 0.5 mm posterior and 1.2 mm lateral to Bregma. A 28G cannula was then inserted 3.0 mm ventral to the skull surface and fixed in place by dental acrylic. The minipump was carefully inserted into a subcutaneous pocket between the scapulae, the skin incision sutured, and the mouse allowed to recover in an incubator until independently mobile. Following standard postoperative care procedures, the mice were then housed individually for the duration of the study.

Postoperative behavioral testing on the OiP associative recognition task occurred 9 days after surgery and used the procedure described in experiments 1 and 2. On day 14, following behavioral testing, the animals were culled and brain tissue collected for analyses.

### 2.5 Ro25-6981 administration

Ro25-6981 (Tocris, Abingdon, UK) was dissolved in sterile saline at a final concentration of 5 mg/mL. Mice were administered a single 10 mg/kg dose of Ro25-6981 or vehicle IP, 30 minutes before the start of the associative recognition OiP task. This dose was selected based on previously published behavioral work (e.g., Mikics et al., 2017).

### 2.6 Pathology: protein extraction and immunoblots

The hippocampus and cortex were dissected, snap-frozen in liquid nitrogen, and stored at −80 °C. Soluble proteins were extracted from brain samples as previously described (Thomas et al., 2011a). For the ICV study, all blots quantifying changes in soluble protein levels for the ICV study assessed 10 WT vehicle, 11 PDAPP vehicle, and 10 PDAPP mice administered 2B3 intracerebroventricularly. For the IP study analysis, 9 WT vehicle, 10 PDAPP vehicle, and 9 PDAPP 2B3-administered mice were assessed. Synaptosome extractions were performed using Syn-PER synaptic protein extraction reagent (Thermo Fisher, UK). Western blotting was performed using standard methods as described previously (Thomas et al., 2011a) using samples from all animals undergoing treatment and behavioral testing. Briefly, after protein quantification, 20 μg of protein/sample was resolved on either a 10% or 7.5% polyacrylamide gel and detected with the relevant antibody (Table 1). The right hippocampus of all 10 WT vehicle, 11 PDAPP vehicle, and 10 PDAPP mice administered 2B3 intracerebroventricularly were used to assess synaptic protein changes.

**Table 1:** Primary antibodies used in Western blotting

Primary antibody	Species	Dilution	Source
APP (22C11)	Mouse	1:1000	Millipore
BACE1	Rabbit	1:1000	Cell Signalling Technology
NMDA-NR1	Mouse	1:1000	BD Biosciences
NMDA-NR2B	Rabbit	1:500	Millipore
NMDA-NR2B pY1472	Rabbit	1:750	Millipore
PSD95	Rabbit	1:1000	Abcam
PS1	Rabbit	1:500	Santa Cruz
Fyn	Mouse	1:750	BD Biosciences
STEP	Mouse	1:750	Novus
ERK	Rabbit	1:2000	Cell Signalling Technology
Phospho-ERK	Rabbit	1:1000	Cell Signalling Technology
GAPDH	Pre-conjugated	1:50,000	Sigma
β-Actin	Pre-conjugated	1:20,000	Sigma

Key: ERK, extracellular signal–regulated kinase; NMDA, N-methyl-D-aspartate; STEP, striatal-enriched phosphatase.

### 2.7 Sandwich ELISA for detection of APP metabolites

ELISAs for the quantification of Aβ were carried out as previously described (Thomas et al., 2011a, Thomas et al., 2011b), or as recommended by the manufacturer: APP (R&D Systems, Abingdon, UK), β-amyloid derived C-terminal fragment (βCTF; IBL, Hamburg, Germany), and Aβ40 and Aβ42 (human and mouse; Invitrogen, California, USA). Data are presented as ng or pg/mg total protein concentration. With exception of experiment 2b, which assessed endogenous mouse Aβ40 and Aβ42, all further ELISAs were sensitive to human protein only. Therefore, the PDAPP mice that expressed the human APPV717F were used. The ICV study used 11 PDAPP vehicle mice and 10 PDAPP 2B3 mice (however, 1 mouse was removed as an outlier as explained below). The IP study used 10 PDAPP vehicle mice and 9 PDAPP 2B3 mice to quantify protein levels by ELISA.

### 2.8 Statistical analyses

All statistical analyses were performed using IBM SPSS statistics. The behavioral data conformed to the assumptions of analysis of variance (ANOVA) and were analyzed using a mixed measures design. Significant interactions were assessed using tests for simple main effects. Western blot data were analyzed using one-way ANOVA followed by Tukey's post hoc analysis. WT data were collapsed across treatment (untreated and vehicle) for the analysis of the cognitive and behavioral effects of 2B3 administration as no group differences were observed between WT groups *p*’s > 0.5 (data not shown). ELISA data were analyzed using either Student’s *t*-test or a Kruskal-Wallis test with Dunn’s test along with a Bonferroni correction for multiple comparisons. All data were subject to Levene’s and Shapiro-Wilks’ tests for data normality before analysis. Appropriate transformations were carried out when necessary.

Data generated from ELISA assays were quantified by comparing data to standard curves from each plate using GraphPad Prism 4 and normalized to total protein concentration. One PDAPP 2B3-treated mouse was found to be an extreme outlier across the ELISA analyses of the 2B3 ex vivo tissue, as determined by SPSS Tukey box plots and more conservative methods for labeling outliers, as described previously (Carling, 1998, Hoaglin and Iglewicz, 1987). No transformation was able to normalize this outlier for either parametric or nonparametric analysis. Therefore, the data from this 1 mouse were removed from all ELISA analyses but no other analysis. The result of this exclusion was that all remaining ELISA data sets were normally distributed, and its exclusion did not change the pattern of results. Western blots were quantified using Image J software (www.imagej.nih.gov/).

## 3 Results

Experiment 1 used object novelty and OiP tasks to determine the nature and age of onset of memory deficits in PDAPP mice. In the second stage of the study, experiment 2a, the same animals were administered the APP antibody 2B3, at 17-18 months of age, via osmotic minipumps for a period of 14 days. Having established that 2B3 reversed pre-existing associative recognition memory impairment, experiment 3 determined whether longitudinal peripheral administration of 2B3 prevented the onset of cognitive decline in PDAPP mice.

### 3.1 Experiment 1

During development, WT mice showed a greater overall object contact time during sample phases across both tasks and at all ages, than PDAPP mice, *p*’s < 0.01 (Supplementary Table 1). However, analysis showed that both WT and PDAPP mice habituated normally across the 3 sample phases, as indicated by a decline in object contact times (Supplementary Table 1). In the test phase, WT mice showed a greater contact time with objects than PDAPP mice, *p* < 0.001 across all ages (Supplementary Table 2). However, both WT and PDAPP mice showed a preference to explore objects in novel locations more than familiar locations both at 6-8 and 10-12 months of age, *p* values <0.01. However, at 14-16 months of age, only WT but not PDAPP mice showed this preference, (*p* < 0.001 and *p* > 0.05, respectively; data collapsed across both delays). There were no significant interactions involving genotype and delay for either task (see Supplementary Analysis). In contrast, both WT and PDAPP mice showed a preference to explore novel objects across all ages and delays, *p* values <0.01, and showed no genotype difference, even at 14-16 months of age (*p* > 0.05; Supplementary Table 2).

**Figure 3:**
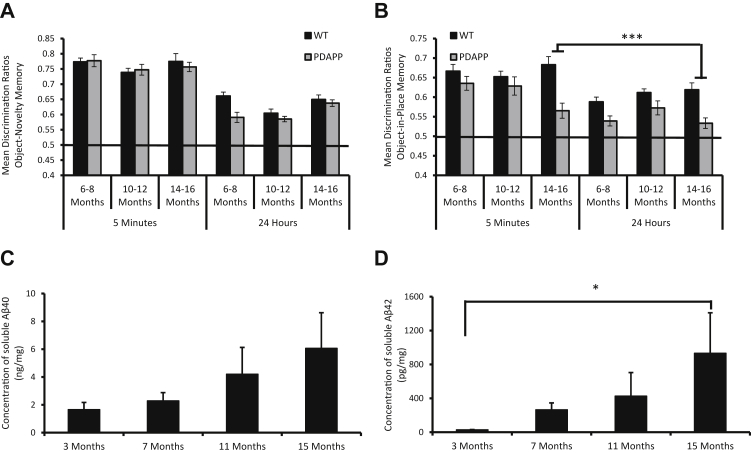
PDAPP mice show an age-dependent decline in OiP memory performance and an increase in amyloid levels. (A) PDAPP mice (n = 14) showed no deficits in object novelty memory across any age or delay compared to WT littermates (n = 15). (B) However, a significant age vs genotype interaction revealed that PDAPP mice showed an age-dependent impairment in OiP memory at 14-16 months of age across both delays compared to WT littermates. There was also a significant decline in the OiP performance of PDAPP mice at 14-16 months of age compared to their performance at 6-8 and 10-12 months of age. DR scores were analyzed using 3-way ANOVA with Bonferroni corrected post hoc analysis for significant interactions. (C-D) Levels of soluble Aβ40 and Aβ42 were then assessed in a separate colony of mice at 3 months (n = 5), 7 (n = 7), 11 (n = 7), and 15 months (n = 7) of age. (C) Levels of soluble Aβ40 and (D) Aβ42 increased with age in the hippocampus of PDAPP mice. Aβ levels were quantified by ELISA assays. Data were analyzed using the Kruskal-Wallis test with Dunn’s post hoc analysis with Bonferroni corrections. *p < 0.05; ***p < 0.001. Error bars represent the standard error of the mean (SEM). Abbreviations: ANOVA, analysis of variance; DR, discrimination ratio; OiP, object-in-place; WT, wild type.

An analysis of DRs similarly showed an identical pattern with PDAPP mice performance, as well as WT mice, on the novelty test at all ages as indicated by no main effect of genotype, *F*(1, 27) = 2.5, *p* > 0.1, and no genotype × age interaction, *F*(1, 27) = 2.8, *p* > 0.1. In contrast, PDAPP mice were significantly impaired relative to control mice only at 14-16 months of age for the OiP task (see Fig. 3A and 3B). Analysis of the OiP task revealed a significant age × genotype interaction, *F*(2, 54) = 9.2, *p* < 0.05. Subsequent tests of simple main effects revealed that PDAPP mice showed a significant main effect of age with a reduction in OiP memory performance across age ranges, *F*(2, 26) = 9.8, *p* < 0.001. Further analysis revealed that PDAPP mice showed a significant memory deficit compared with WT mice only at 14-16 months of age, *F*(1, 27) = 49.9, *p* < 0.001. There was no significant interaction involving delay, *F* < 1. The analysis of DR values confirmed that PDAPP mice showed an age-dependent deficit in associative recognition memory, without affecting object novelty/familiarity discriminations.

An analysis of age-related changes in hippocampal Aβ levels in a separate group of PDAPP mice confirmed a numerical 3.6-fold increase in soluble Aβ40 levels (Fig. 3C; nonsignificant following analysis by the Kruskal-Wallis test, X^2^(3) = 2.7, *p* > 0.1) and a significant, X^2^(3) = 10.5, *p* < 0.05, 32-fold increase in soluble Aβ42 levels by 15 months of age (Fig. 3D). Dunn’s test for multiple comparisons showed a significant increase in the levels of soluble Aβ42 when comparing mice at 3 months and 15 months of age, *p* < 0.05. Thus, PDAPP mice showed an age-related increase in amyloid pathology with a marked increase in Aβ production at the same age as behavioral deficits emerged in a separate cohort of mice.

### 3.2 Experiment 2

#### 3.2.1 Experiment 2a

After 2B3 or vehicle administration, PDAPP mice continued to explore all 4 objects less than WT mice, *p* values <0.01. However, all mice continued to show reduced contact times across sample trials, *p*’s < 0.001 (Supplementary Table 3A). During the test phase, overall mice showed a preference to explore objects in novel locations over familiar, *p* < 0.001 (Supplementary Table 4A). However, WT mice had higher contact times with objects both in familiar and novel locations than vehicle PDAPP mice, *p* values <0.01, and PDAPP 2B3-treated mice, *p* values <0.05. No significant differences in contact times were observed between PDAPP treatment groups, *p* > 0.1.

**Figure 4:**
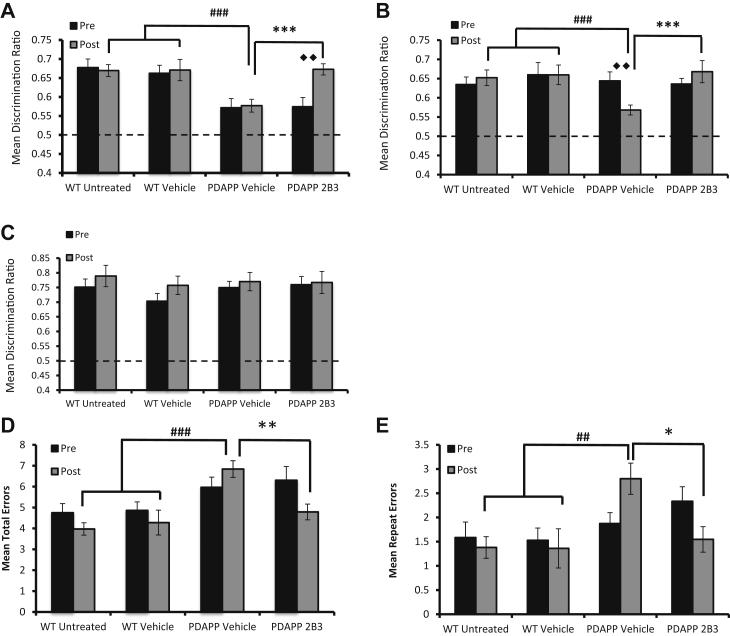
2B3 administration prevents the onset of and reverses age-dependent cognitive deficits in PDAPP mice. (A) PDAPP mice treated with 2B3 (n = 10) by ICV administration showed a significant improvement in OiP memory compared with their pretreatment scores (◆◆*p* < 0.01) and performed significantly better than vehicle-treated PDAPP mice (n = 11; ***p < 0.001). No difference in DR scores were reported when comparing WT mice (untreated n = 11, vehicle n = 10) to PDAPP 2B3 mice. However, vehicle PDAPP mice remained impaired compared with WT mice (###*p* < 0.001). (B) Peripheral administration of 2B3 to PDAPP mice (n = 9) before the onset of impaired OiP performance showed that no decline in OiP performance occurred after 15-week 2B3 administration, *p* > 0.05. 2B3 PDAPP mice further showed a greater OiP performance than vehicle PDAPP mice (n = 10), (***p < 0.001), which did show an age-dependent decline in OiP performance (◆◆*p* < 0.01). PDAPP vehicle mice were further impaired compared with WT mice (untreated n = 10, vehicle n = 10; ###*p* < 0.001), whereas 2B3-administered PDAPP mice were not. (C) No significant differences were reported between groups in peripheral 2B3 administration when comparing novel object recognition memory, indicating all mice were able to encode object information. (D) Peripheral PDAPP vehicle mice showed an age-related increase in foraging errors compared with WT mice (###*p* < 0.001). PDAPP mice administered 2B3, however, made less foraging errors than PDAPP vehicle controls (**p < 0.01) and less errors than their pre-2B3 administration scores (◆*p* < 0.05). (E) Peripheral vehicle PDAPP mice showed an age-dependent increase in repeat errors made in the foraging task (◆*p* < 0.05), which was not observed following 2B3 administration (*p* > 0.05). Vehicle PDAPP mice made significantly more repeat errors than 2B3 PDAPP mice (*p < 0.05) and WT littermate controls (##*p* < 0.01). DR scores and foraging errors were analyzed using a 3-way ANOVA with Bonferroni corrected post hoc analysis for significant interactions. Error bars express the SEM. Abbreviations: ANOVA, analysis of variance; DR, discrimination ratio; ICV, intracerebroventricular; OiP, object-in-place; SEM, standard error of the mean; WT, wild type.

An analysis of the DRs, however, showed a significant treatment × time point interaction, *F*(2, 39) = 3.27, *p* < 0.05. Tests of simple main effects revealed that PDAPP mice administered ICV 2B3 showed a significant improvement in performance compared to both their pre-administration, *p* < 0.01, and to vehicle PDAPP mice performance, *p* < 0.001. In contrast, vehicle PDAPP mice remained significantly impaired relative to WT controls, *p* < 0.001, and 2B3 PDAPP mice were not significantly different to WT mice, *p* > 0.5 (see Fig. 4A). Thus, ICV infusion of 2B3 in PDAPP mice reversed an age-dependent deficit in associative recognition memory.

#### 3.2.2 Experiment 2b

To investigate further how inhibition of APP metabolism at the BACE1 cleavage site affected healthy WT control animals, a separate group of mice were directly administered ICV 2B3. Similar to PDAPP mice, 2B3 administration in WT mice showed no overall changes in object contact times across sample phases for object novelty and OiP tasks relative to WT IgG1κ controls, *p*’s > 0.1 nor during the test phases, *p*’s > 0.1 (Supplementary Table 3C and 4C, respectively). Interestingly, while both 2B3 and control WT IgG1κ mice explored novel objects in preference to familiar objects, a numerical reduction in contact times with objects in novel locations was observed in 2B3 mice (Supplementary Table 4C); however, this failed to reach statistical significance. When the data from both tasks were converted to DRs, there was no significant difference between 2B3 and control IgG1κ mice on the novelty test (t < 1) but a significant deficit in 2B3 mice on the OiP task [t(13) = 2.66, *p* < 0.05; Supplementary Figure 1B]. Furthermore, both groups performed the novel object recognition memory task above chance (0.5), *p*’s < 0.01 (Supplementary Figure 1A). In contrast, 2B3 mice did not perform above chance level (0.5) on the OiP task, *p* > 0.5, unlike WT IgG1κ control mice, *p* < 0.01 (Supplementary Figure 1B). These results indicate that 2B3 (but not a control IgG1κ) administered to normal WT mice leads to a selective disruption of associative recognition memory.

### 3.3 Experiment 3

Analysis of contact times with objects across sample trials for both recognition memory tasks showed that all groups explored the objects at similar levels, *p* > 0.05 (Supplementary Table 3B). All mice also showed habituation of exploratory activity to objects, as indicated by a decrease in contact times when comparing sample trial 1 to sample trial 3, *p*’s < 0.001. Following contact time analysis within the test phase of the object novelty and OiP tasks, mice showed no significant differences in overall contact times with all objects *p*’s > 0.05 (Supplementary Table 4B). In the OiP task, despite treatment groups showing numerical differences in contact times between objects in novel and familiar locations, all mice continued to show a general preference to explore objects in novel locations over familiar, *p* < 0.001. The same pattern was also observed in the object novelty task.

However, analysis of the OiP test DRs revealed a significant time × treatment group interaction, *F*(2, 34) = 3.41, *p* < 0.05. Testing for simple main effects further revealed that, although vehicle PDAPP mice showed a decline in OiP performance across pre- and post-treatment time points, *p* < 0.01, IP administration of 2B3 prevented this decline, *p* > 0.05. Moreover, both WT and 2B3 PDAPP mice showed comparable performance (*p* > 0.05) and both groups performed better than vehicle PDAPP mice, *p* values <0.001 (Fig. 4B). Analysis of the object novelty task DRs showed no effect of treatment group, *F*(2, 34) = 0.16, *p* > 0.1, or treatment group × time interaction, *F*(2, 34) = 0.27, *p* > 0.1, (Fig. 4C). To summarize, the results showed that peripheral administration of 2B3 before the onset of cognitive decline prevented the age-dependent deficit in associative recognition, without influencing object novelty detection, in PDAPP mice.

To establish the generality of the improvement in associative OiP memory with 2B3, PDAPP mice were also tested on a spatial working memory foraging task (Evans et al., 2018). Time to complete the foraging task after vehicle or 2B3 administration was similar between all groups, *p* > 0.05 (Supplementary Table 5), which indicated that neither transgene expression nor 2B3 administration influenced the motivation to complete the task. However, foraging accuracy was improved in 2B3 PDAPP relative to vehicle PDAPP mice (Fig. 4D and E). More specifically, analysis of the mean total number of errors (a measure of overall foraging accuracy) revealed a significant treatment time × group interaction, *F*(2, 34) = 3.68, *p* < 0.05. Tests of simple main effects showed that 2B3 PDAPP mice made less errors than their pre-drug assessment at 11 months of age, *p* < 0.05 and less errors than vehicle PDAPP mice, *p* < 0.01 (Fig. 4D). In contrast, vehicle PDAPP mice made more errors than WT vehicle controls, *p* < 0.001 (Fig. 4D). WT mice and 2B3 PDAPP mice performed at a comparable level, *p* > 0.05. A similar pattern of results was observed when analyzing repeat errors, a measure of working memory (Fig. 4E). An ANOVA revealed a significant treatment time × group interaction, *F*(2, 34) = 3.85, *p* < 0.05. Simple main effects analysis revealed that 2B3 PDAPP mice made fewer repeat errors than vehicle PDAPP mice, *p* < 0.05, and that there was no significant change in the number of repeat errors compared to pre-drug performance, *p* > 0.05. In contrast, vehicle PDAPP mice showed an age-dependent increase in the total number of repeat errors compared to pre-drug assessment, *p* < 0.05. Vehicle PDAPP mice also made more repeat errors than WT mice, *p* < 0.01. This impairment was not present in 2B3 PDAPP mice, *p* > 0.05.

### 3.4 Biochemical analyses: experiments 2a, 2b, and 3

**Figure 5:**
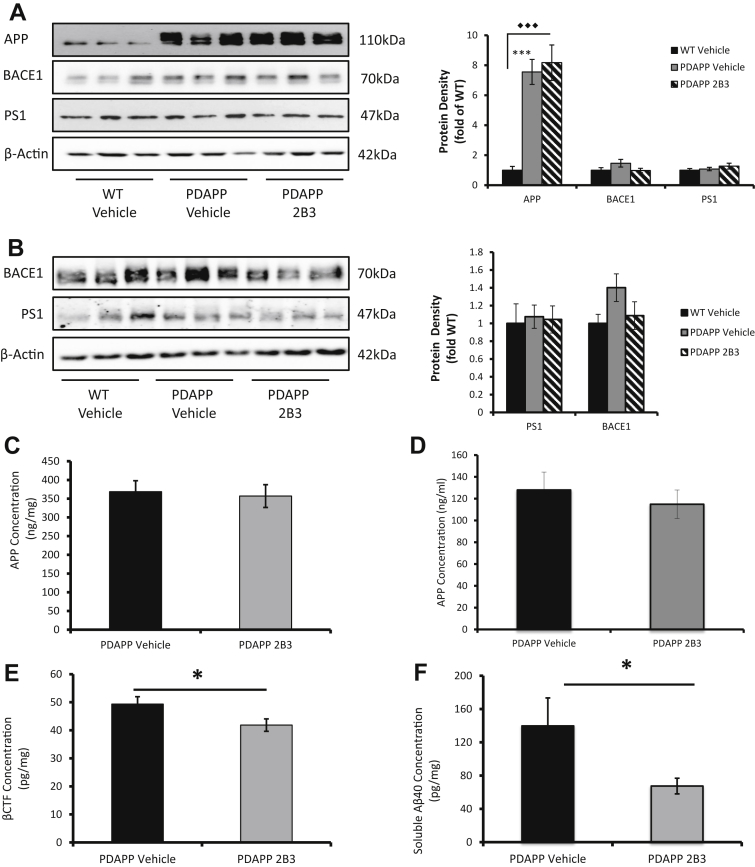

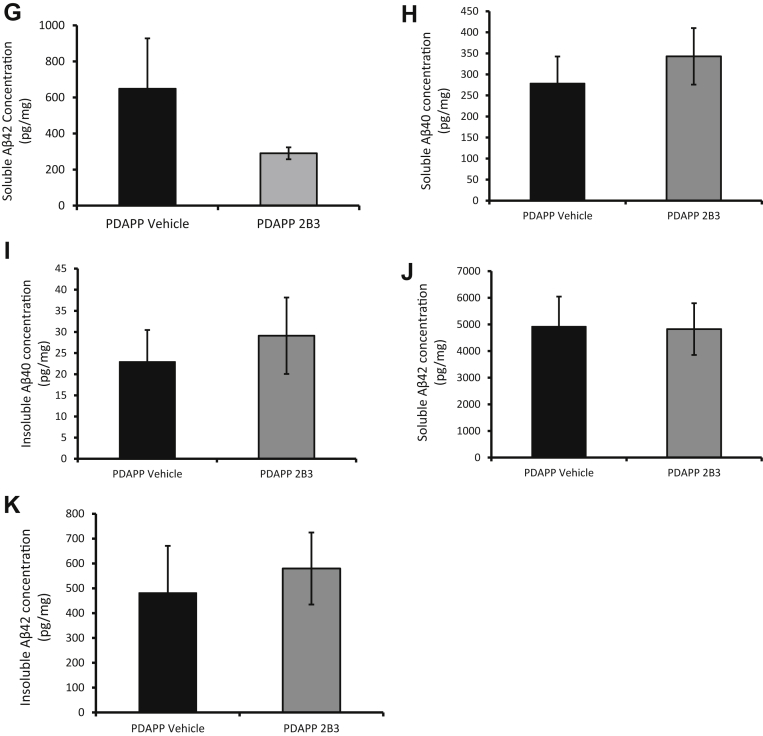
2B3 inhibits APP metabolism by BACE and reduces Aβ production. (A) 2B3 administration by ICV in PDAPP mice (n = 10) showed no overall changes in total levels of BACE1 or PS1 in the hippocampus compared with PDAPP vehicle mice (n = 11) or WT vehicle mice (n = 10), *p*'s > 0.5, as determined by Western blot. However, ICV vehicle and 2B3 PDAPP mice showed a significant increase in total levels of APP (***p < 0.001, ◆◆◆*p* < 0.001), respectively, relative to WT littermates as determined by one-way ANOVA with post hoc Tukey analysis. (B) Similar to ICV mice, after IP administration of 2B3 to PDAPP mice (n = 9), no change in BACE1 or PS1 was observed compared with vehicle PDAPP (n = 10) or WT mice (n = 9). (C and D) When protein levels were quantified using ELISA, PDAPP mice administered with 2B3 showed no change in total levels of APP after ICV (n = 9) or IP (n = 9) administration of 2B3 compared with vehicle PDAPP mice (ICV n = 11, IP n = 10). (E) ICV 2B3 administration caused a significant reduction in βCTF, and (F) soluble Aβ40 and (G) a nonsignificant reduction in soluble Aβ42 in the hippocampus of PDAPP mice. (H-K) No significant changes in amyloid levels were observed in IP 2B3-administered mice compared with vehicle PDAPP mice. Data were analyzed using independent samples t-tests. *p < 0.05. Error bars express the SEM. Abbreviations: ANOVA, analysis of variance; DR, discrimination ratio; ICV, intracerebroventricular; IP, intraperitoneal; OiP, object-in-place; SEM, standard error of the mean; WT, wild type.

*In experiment 2a,* Western blot analysis showed that ICV 2B3 administration caused no change in total levels of BACE1, *F*(2, 28) = 1.95, *p* > 0.1, or presenilin 1 (PS1), *F*(2, 28) = 1.40, *p* > 0.1, in the hippocampus of PDAPP mice (Fig. 5A). These data were replicated in the hippocampus of IP 2B3 mice for both BACE1, *F*(2, 25) = 2.30, *p* > 0.1, and PS1, *F* < 1 (Fig. 5B), and WT ICV 2B3 mice for BACE1, t(13)=0.5, *p* > 0.5 (Supplementary Figure 1D). Western blot analysis showed an overall change in APP levels, *F*(2, 28) = 8.49, *p* < 0.01 in ICV 2B3 PDAPP mice (Fig. 5A). Post hoc Tukey tests revealed this difference was evident only when comparing WT mice to PDAPP vehicle, *p* < 0.01, and PDAPP 2B3 mice, *p* < 0.05. No difference in APP levels was reported between the PDAPP groups, *p* > 0.1. ELISA analysis further confirmed that 2B3 caused no change in APP levels in PDAPP mice after either ICV, t(18) = 1.32, *p* > 0.1, or IP administration, t(17) = 1.45, *p* > 0.1 (Fig. 5C and D, respectively). In experiment 2b, there was similarly no change in endogenous APP levels in WT mice administered 2B3, relative to IgG1κ controls t(13) = 0.81, *p* > 0.5 (Supplementary Figure 1D).

In experiment 2a, ICV 2B3 administration caused a significant 16% reduction in levels of βCTF, t(18) = 2.22, *p* < 0.05, a significant 56% reduction in levels of soluble Aβ40, t(18) = 2.28, *p* < 0.05, and a (nonsignificant) 66% reduction in levels of soluble Aβ42, t(18) = 1.01, *p* > 0.1, in the hippocampus (Fig. 5E, F, and G, respectively; see also in vitro data from Thomas et al., 2011b, Thomas et al., 2013). In experiment 2b, there was a significant 8.1% reduction in endogenous soluble Aβ40, t(13) = 2.45, *p* < 0.05, in the hippocampus of WT ICV 2B3 mice relative to IgG1κ control mice (Supplementary Figure 1E). Endogenous soluble Aβ42 was undetectable by ELISA and, therefore, could not be compared (data not shown). In experiment 3, analysis of hippocampal tissue from IP 2B3 PDAPP mice revealed no significant change in soluble or insoluble Aβ40 or Aβ42 or βCTF levels (data not shown) compared with vehicle PDAPP mice, t's < 1 (Fig. 5H-K).

**Figure 6:**
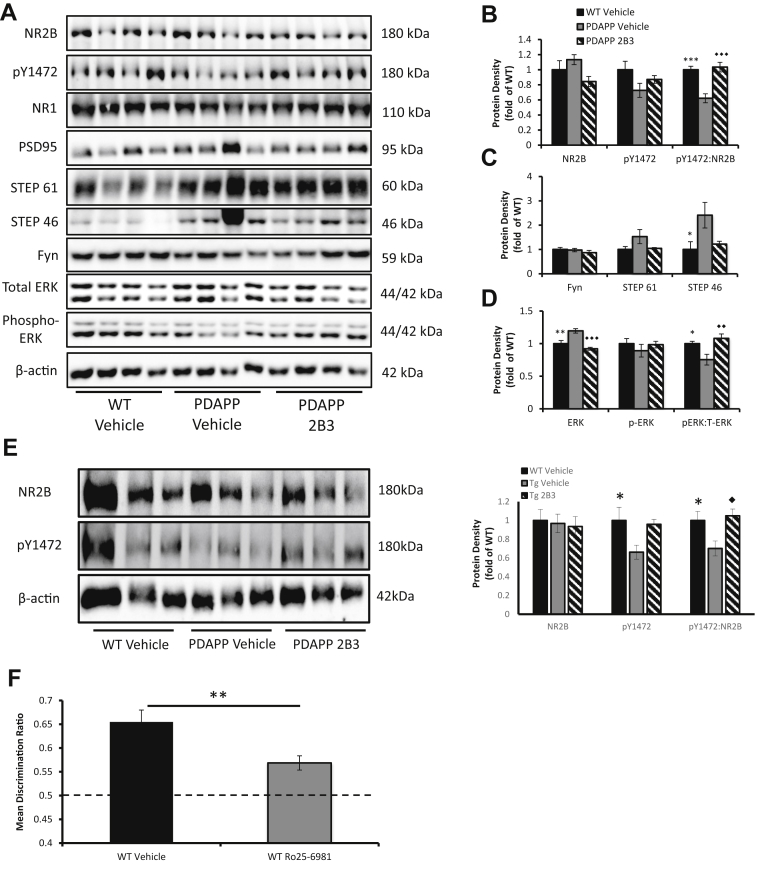
Impaired NMDA-NR2B signaling causes OiP impairment, which is reversed by 2B3 in PDAPP mice. (A) Representative Western blots of hippocampal synaptosomes from PDAPP mice administered 2B3 by ICV (n = 10) or vehicle (n = 11) and WT vehicle control (n = 10). Blots demonstrate changes in NR2B phosphorylation and subsequent downstream signaling cascades. No changes in total levels of PSD95, NR1 (total NMDA receptors) (relative to WT control) were reported *p* > 0.05. (B) No significant changes were reported in total levels of NR2B or pY1472, *p*'s > 0.05. However, when pY1472 was expressed as a ratio of total NR2B, PDAPP vehicle mice showed a significant reduction in NR2B phosphorylation compared with WT (***p < 0.001) and 2B3 PDAPP mice (◆◆◆*p* < 0.001). (C) No significant changes were reported in total levels of the Src kinase Fyn, or STEP61 (*p* values > 0.05); however, PDAPP vehicle mice showed a significant increase in total levels of STEP46 compared with WT mice (*p < 0.05). (D) Total levels of ERK were increased in vehicle PDAPP mice compared with WT (**p < 0.01) and 2B3 PDAPP mice (◆◆◆*p* < 0.001). Despite an apparent numerical reduction in phosphorylated (active) ERK in vehicle PDAPP mice, no significant differences were reported (*p* values > 0.05). However, when phosphorylated ERK was expressed as a ratio of total ERK, vehicle PDAPP mice had a significant reduction in phosphorylated ERK compared with WT (*p < 0.05) and 2B3 PDAPP mice (◆◆*p* < 0.01). (E) A similar result was observed in PDAPP mice administered chronically with 2B3 by IP injection. A representative blot demonstrates a reduction in NR2B pY1472. No significant change was observed in total levels of NR2B (*p* values >0.1). Vehicle PDAPP mice showed a significant reduction in pY1472 compared with WT controls (*p < 0.05). When pY1472 was expressed as a ratio of total NR2B, vehicle PDAPP mice (n = 10) showed a significant reduction in comparison to WT control (n = 9; *p < 0.05) and 2B3 PDAPP mice (n = 9; ◆*p* < 0.05). There was no significant difference when comparing WT and 2B3 mice. (F) DR scores of the OiP task after Ro25-6981 administration, a selective NR2B antagonist, showed impaired OiP performance in 17- to 18-month-old WT mice (n = 10) compared with vehicle performance (**p < 0.01). All Western blot data were analyzed using 1-way ANOVA with Tukey post hoc analysis. DR scores were analyzed using paired-samples t-tests. Error bars represent the SEM. Abbreviations: ANOVA, analysis of variance; DR, discrimination ratio; ERK, extracellular signal–regulated kinase; ICV, intracerebroventricular; IP, intraperitoneal; OiP, object-in-place; SEM, standard error of the mean; WT, wild type.

In experiment 2a, there were no significant differences in the total expression levels of the synaptic marker PSD95 or NMDA receptor subunits, NR1, or NR2B in hippocampal synaptosomes of ICV [maximum NR2B, *F*(2, 28) = 2.72, *p* > 0.05]. A similar pattern was evident in experiment 3 after IP 2B3 administration (maximum NR1, *F*(2, 26) = 0.12, *p* > 0.1, respectively; Fig 6A, B and E). In experiment 2a, phosphorylation of the NR2B Y1472 residue appeared to be reduced in vehicle PDAPP mice, although this was not significant relative to WT mice or ICV 2B3 PDAPP mice, *F*(2, 28) = 2.36, *p* > 0.05 (Fig. 6A and B). However, after IP administration of 2B3 (experiment 3), phosphorylation of the NR2B Y1472 residue was significantly altered in IP 2B3 PDAPP mice, *F*(2, 25) = 3.86, *p* < 0.05, PDAPP vehicle mice showed reduced pY1472 compared with WT vehicle mice, *p* < 0.05 (Fig. 6E). When NR2B Y1472 phosphorylation was presented as a ratio of total NR2B, there was a significant difference between groups in both the ICV (experiment 2a), *F*(2, 28) = 16.11, *p* < 0.001 and IP (experiment 3) studies, *F*(2, 25) = 5.30, *p* < 0.01. Post hoc Tukey's analyses revealed a reduction in vehicle PDAPP mice compared with WT mice in both ICV and IP groups (*p* < 0.001, *p* < 0.05; Fig. 6B and E). Furthermore, the ratios were also reduced in vehicle PDAPP mice compared with 2B3 PDAPP mice, (*p* < 0.001, *p* < 0.05, respectively). Neither ICV nor IP 2B3 PDAPP mice differed relative to their respective WT control mice, *p* values > 0.1.

Given the changes in NR2B phosphorylation, we investigated SRC kinase Fyn and striatal-tyrosine-enriched phosphatase (STEP) in hippocampal synaptosomes of ICV 2B3 PDAPP mice. These kinases are key regulators of NR2B phosphorylation (Ittner et al., 2010, Zhang et al., 2010). No change in total levels of Fyn between any of the groups was evident in hippocampal synaptosomes, *F* < 1 (Fig. 6C). Conversely, both isoforms of STEP (46 and 61) were numerically increased in vehicle PDAPP mice relative to WT and 2B3 PDAPP mice (Fig. 6C). However, despite a numerical increase in STEP61, there were no significant differences between groups, *F*(2, 28) = 2.48, *p* > 0.05. In contrast, STEP46 was altered significantly, *F*(2, 28) = 4.39, *p* < 0.05. Post hoc Tukey's analysis revealed STEP46 was increased in vehicle PDAPP mice compared with WT mice, *p* < 0.05 (there was a numerical difference between PDAPP vehicle and 2B3 PDAPP mice, but it was not significant; *p* = 0.074, 2B3; Fig. 6C).

Extracellular signal–regulated kinase (ERK) is downstream of NMDA signaling and plays a role in regulating memory processes (Caccamo et al., 2010). There was a significant change in total ERK levels [*F*(2, 28) = 15.29, *p* < 0.001] in synaptosomes of ICV vehicle PDAPP compared with WT (*p* < 0.01) and ICV 2B3 PDAPP mice (*p* < 0.001; Fig. 6D). There were no changes between ICV WT and 2B3 PDAPP mice in total levels of ERK, *p* > 0.1. Although phosphorylated ERK appeared to be numerically reduced in ICV vehicle PDAPP mice, there were no significant differences between groups, *F* < 1. In contrast, when pERK was expressed as a ratio of total ERK, to better determine ERK activity, ICV vehicle PDAPP mice showed a significant difference, *F*(2, 28) = 7.03, *p* < 0.01, with a significant reduction compared with both WT (*p* < 0.05) and ICV 2B3 PDAPP mice (*p* < 0.01). There was no significant difference between WT and ICV 2B3 PDAPP mice, *p* > 0.05 (Fig. 6D).

### 3.5 Experiment 4

The aforementioned analyses suggested that the NR2B receptor played an important role in associative recognition memory, although there are relatively little published data to support this conclusion. We therefore tested a separate group of 18-month-old WT mice on the OiP task after the administration of the NR2B receptor antagonist, Ro25-6981. WT mice continued to show habituation to objects across 3 sample phases after Ro25-6981 or vehicle administration (Supplementary Table 6). Ro25-6981 did not change overall object contact times of WT mice in either the sample or test phases when compared with vehicle contact times, *p*'s > 0.05 (Supplementary Tables 6 and 7). Moreover, despite numerical differences, both vehicle (*p* < 0.001) and Ro-25-6981 (*p* < 0.01) mice explored objects in novel locations more than those in familiar locations (Supplementary Table 7). However, an analysis of the test DR scores showed that Ro25-6981 impaired performance relative to vehicle administration, t(9) = 2.47, *p* < 0.05 (Fig. 6F). These data confirm that, under normal physiological conditions, the NR2B receptor plays a key role in associative recognition memory processes in normal aged WT mice.

## 4 Discussion

Male PDAPP mice showed a selective age-dependent impairment in associative recognition memory, while sparing object novelty detection. Similar to previous studies, the age-dependent decline in associative recognition memory coincided with a rise in hippocampal Aβ levels (Barker and Warburton, 2013, Good and Hale, 2007, Hale and Good, 2005, Selkoe, 2001). ICV administration of the anti-APP antibody, 2B3, directed against the BACE1 cleavage site, reduced APP metabolism and lowered Aβ and βCTF levels in the hippocampus. The reduction in Aβ was accompanied by restoration of associative recognition memory in aged PDAPP mice. Furthermore, longitudinal peripheral administration of 2B3 prevented the onset of associative recognition memory impairment as well as a decline in spatial working memory in PDAPP mice at 15 months of age. A critically important aspect of our results is that both ICV and IP administration of 2B3 normalized a deficit in NMDA receptor phosphorylation. Previous work in normal animals has shown that hippocampal NMDA receptors are required for associative recognition memory but not object novelty/familiarity discriminations (Barker and Warburton, 2013). Our results are, therefore, consistent with the view that the age-related accumulation of Aβ changes hippocampal synaptic events that underpins memory (Guntupalli et al., 2016).

Previous work with PDAPP mice has reported an age-related deficit in object novelty detection (Dodart et al., 1999). However, this result has not been replicated across laboratories (Chen et al., 2000). This discrepancy may be related to the test procedure. In contrast to our own study and that conducted by Chen et al., Dodart et al. (1999) exposed mice to a single object in the sample phase and presented a familiar and novel object in the test phase. This test procedure confounded object novelty with object-location novelty. Thus, the deficit in PDAPP mice reported by Dodart et al. may have reflected impaired processing of location information. Importantly, with the exception of the present experiments, no study has compared the impact of aging on object novelty and associative OiP memory in the same PDAPP mice longitudinally, and our evidence indicates an age-related sensitivity to associative object-location memory or mismatch detection in PDAPP mice.

Before discussing the effects of 2B3 on APP processing and cognition, it is worth highlighting 2 drawbacks of the study. One drawback is that only male mice were tested. This strategy was undertaken to minimize extraneous sources of variability in both the behavioral and biological measures and thus maximize the detection of cognitive and synaptic changes induced by 2B3. Nevertheless, given the positive results of this study in male mice and the fact that over 60% of patients with AD are female (“2015 Alzheimer’s disease facts and figures,” 2015), it would be important clearly to test the assumption that aged female PDAPP mice would also demonstrate cognitive and pathology benefits from reducing APP cleavage by BACE1.

A second drawback of the study is that the half-life of 2B3 has yet to be established in vivo. Although a single weekly injection of 2B3 was sufficient to alter NR2B expression and cognition in PDAPP mice, there was no evidence of changes in brain amyloid levels, unlike the ICV administration study. However, this lack of sensitivity to any changes probably reflects the much lower levels of 2B3 reaching the brain after peripheral IP administration, (because of the blood-brain barrier) compared with direct ICV administration. Further research is required to assess the temporal dynamics of the interactions of 2B3 with APP processing in vivo. Nevertheless, our data present clear evidence that, even under a restricted set of conditions, 2B3 has an impact on APP processing and memory.

Excess Aβ production leads to changes in the dynamic properties of hippocampal synaptic plasticity; promoting the induction of LTD and impairing the induction of LTP (Hsieh et al., 2006). This change in plasticity dynamics occurs through several mechanisms involving NMDA receptors and intracellular calcium signaling. Indeed, extra-synaptic NMDA-NR2B receptors have emerged as a key factor in mediating the effects of amyloid on synaptic depression and toxicity (Kessels et al., 2013, Wang et al., 2013); for example, low nanomolar levels of soluble Aβ oligomers enhance NR2B-mediated NMDA receptor currents and extra-synaptic responses (Li et al., 2011).

Excess Aβ production can also result in activation of Fyn kinase, which leads to phosphorylation of NR2B at Y1472 and stabilizes expression of the receptor at the membrane surface. The effect of Aβ on Fyn kinase is counteracted by elevation of STEP (Ittner et al., 2010, Zhang et al., 2010); for example, there is an age-related increase in STEP levels in Tg2576 mice (Zhang et al., 2010). STEP, a tyrosine phosphatase, dephosphorylates NR2B at the Y1472 site, as well as Fyn kinase and AMPA receptors. STEP therefore controls NMDA receptor activity by directly dephosphorylating NR2B and deactivating Fyn kinase. The enhanced expression of NR2B seen with Aβ is thought to contribute ultimately to the loss of synaptic connections (Boehm, 2013). In the present study, 2B3 (both ICV and IP) reversed the phosphorylation of NMDA-NR2B pY1472 in hippocampal synaptosomes, without affecting PSD95, NR1, or NR2B expression. We further showed an increase in total levels of STEP in vehicle PDAPP mice, which was numerically reduced after 2B3 administration. Activation of ERK, which is downstream of NMDA receptor activity, plays a role in regulating memory processes (Krapivinsky et al., 2003). Furthermore, dysregulation of NR2B/ERK signaling has been reported in 3xTg mice in association with elevated levels of amyloid (Caccamo et al., 2010). In the present study, ERK activity was reduced when expressed as a ratio of total ERK in hippocampal synaptosomes from PDAPP mice. The reduction in ERK was, however, reversed after 2B3 administration. Taken together, this evidence suggests that the 2B3-mediated changes in NR2B synaptic signaling processes contributed to the improvement in associative recognition memory in aged 2B3 PDAPP mice. In support of this assertion, we showed for the first time that selective antagonism of NR2B receptors impaired associative OiP recognition memory in WT mice.

It is important to acknowledge that other anti-APP-BACE1 and immunization approaches have been investigated. Thus, Arbel et al., (2005) reported the activity of the antibody BBS1 also directed against the BACE1 cleavage site of APP. In vivo administration of BBS1 by osmotic minipumps to 3xTg mice for 28 days resulted in a significant improvement in object-novelty memory at 17-18 month of age (Rabinovich-Nikitin et al., 2012). These improvements in behavior were complemented by a significant reduction in plaque size, Aβ load, and a 24% reduction in soluble Aβ42 (Rabinovich-Nikitin et al., 2012). Peripheral administration of BBS1 also resulted in improved object-novelty memory and reduced inflammatory markers in Tg2576 mice (Rakover et al., 2007) as well as reduced Aβ plaques and intracellular Aβ load in mice with the hAPP V717L London mutation (Arbel-Ornath et al., 2009). Despite the changes in Aβ production, BBS1 did not improve spatial reference memory, as assessed in the water maze. Our current findings confirm that steric hindrance of APP processing by BACE1 can have beneficial effects on amyloid levels and associative spatial recognition memory processes that rely upon the hippocampus. Unlike the present study, however, the effect of BBS1 on synaptic processes sensitive to amyloid accumulation was not examined. A related study by Chang et al., (2007) immunized mice against memapsin-2 (BACE1) with the hypothesis that anti-memapsin-2 antibodies would bind to memapsin-2 located on the surface of neurons and, when endocytosed, would interfere with the cleavage of APP and thus lower Aβ production. Immunization of Tg2576 with anti-M2 saw a reduction in amyloid load and improvement in performance in a water maze reference memory task. Unlike the present study, however, the extent of the cognitive change was not indexed against WT control mice but only vehicle-treated Tg2576 mice. However, taken together, our results, and those of Chang (ibid), confirm that selectively targeting BACE cleavage of APP can have positive effects on amyloid production and memory function. Importantly, however, our study is the first to show that an anti-APP antibody targeting the BACE1 cleavage site improved associative recognition memory and spatial working memory and reduced phosphorylation of NMDA-NR2B receptors. In addition, it is worth noting that 2B3 caused a significant reduction in levels of βCTF, an effect that has not previously been reported with BBS1 or anti-M2. Increased levels of βCTF have been observed in patients with AD, and recent research has revealed an Aβ-independent mechanism causing dysregulation of endocytosis (Kim et al., 2015, Pimplikar et al., 2010). Consistent with this evidence, genetic reduction in BACE1 activity in a mouse model of Down syndrome improved endosomal volume and improved cholinergic markers without lowering Aβ levels (Jiang et al., 2016). It is possible, therefore, that the reduction in βCTF levels by 2B3 may have had a beneficial impact on endocytic pathways, and this possibility requires further investigation.

In terms of the clinical relevance of our findings, evidence is beginning to emerge that some antibody-based therapies have a positive impact on amyloid pathology and may be disease-modifying in amyloid-positive patients (e.g., BAM2401, https://www.eisai.com; aducanumab, Sevigny et al., 2016). Although these findings are preliminary, the idea that such immunotherapies may be useful in the context of treating individuals who are amyloid positive in old age is gaining traction. However, our evidence that 2B3 disrupted associative (but not object novelty) recognition memory in normal young mice confirms that metabolism of APP by BACE1 is an important physiological process that contributes to memory in healthy controls (see also Blume et al., 2018, Ou-Yang et al., 2018; and review by Zhu et al., 2018 for further discussion). Therefore, targeting this pathway in healthy presymptomatic aged participants should be combined with close monitoring for deleterious changes in memory function.

As highlighted by Piton et al. (2018), modulation of BACE activity remains a viable strategy in trials on prodromal and early AD. Our data add to a growing body of preclinical evidence that selectively modifying APP processing at an early stage of amyloid accumulation in the aging amyloid-positive brain has a beneficial effect on cognition. It also confirms that APP processing by BACE1 has a role in normal memory function and that disruption to this equilibrium is detrimental. An antibody therapy that targets APP processing by BACE1 may therefore only have clinical relevance in the context of amyloid-related cognitive changes in the elderly (see Farrell et al., 2017, Hedden et al., 2012).

## Conclusion

In conclusion, the present study has shown that selectively influencing APP metabolism by reducing BACE1 activity by steric hindrance with an antibody, reversed and protected against an age-dependent associative recognition memory deficit in PDAPP mice. ICV administration of 2B3 reduced levels of soluble Aβ and βCTF without affecting total levels of APP, and both ICV and chronic IP administration normalized the phosphorylation of NMDA-NR2B receptors. These novel findings provide important evidence that selective inhibition of APP processing at the BACE1 cleavage site can improve both memory and markers of synaptic pathology in a mouse model of age-related amyloid accumulation. Finally, the results contribute to other findings suggesting that modification of APP processing, and associated downstream glutamatergic signaling cascades, may be beneficial in populations with altered APP metabolism, including aged individuals (Rodrigue et al., 2012) and those at high risk of AD.

## Disclosure statement

All authors declare that they have no competing financial interests.

## Appendix A Supplementary data

NBA (2B3) Supplementary Data (Review 2)_V2
