# Supplementary material for: Selective reduction of APP-BACE1 activity improves memory via NMDA-NR2B receptor-mediated mechanisms in aged PDAPP mice
Source: Neurobiol Aging. 2019 Mar;75:136–49. doi: 10.1016/j.neurobiolaging.2018.11.011 (PMC6357873; doi:10.1016/j.neurobiolaging.2018.11.011)
Supplement: NBA (2B3) Supplementary Data (Review 2)_V2 [file mmc1.docx]

**SUPPLEMENTARY DATA**

**Supplementary Table 1. PDAPP mice show no age-related change in object contact times or short-term habituation to 2 identical (object novelty task) or 4 different objects (object-in-place task) during sample exposure.**

| Age | Genotype | Mean contact time during the sample phases (seconds) with all objects | | |
| --- | --- | --- | --- | --- |
|  |  | Sample Phase 1 | Sample Phase 2 | Sample Phase 3 |
|  | | Object-Novelty Task (2 Identical Objects) | | |
| 6-8 Months | WT | 25.69 | 17.15 | 11.86 |
|  | PDAPP | 18.48 | 12.21 | 8.87 |
| 10-12 Months | WT | 21.40 | 14.20 | 13.64 |
|  | PDAPP | 13.51 | 9.90 | 8.01 |
| 14-16 Months | WT | 25.97 | 13.64 | 9.81 |
|  | PDAPP | 14.60 | 11.01 | 7.81 |
|  | | Object-in-Place Task (4 Different Objects) | | |
| 6-8 Months | WT | 26.91 | 18.21 | 13.64 |
|  | PDAPP | 20.70 | 14.59 | 11.60 |
| 10-12 Months | WT | 36.77 | 21.57 | 14.53 |
|  | PDAPP | 19.40 | 11.99 | 10.62 |
| 14-16 Months | WT | 46.53 | 29.80 | 22.08 |
|  | PDAPP | 21.42 | 16.01 | 11.67 |

Supplementary Table 1: Contact time data (seconds) across 3 sample phases for both object novelty and object-in-place tasks. Contact times are displayed for each age tested and represent the mean contact time of the given sample phase for both delay periods tested.

Recognition memory was tested in PDAPP mice over a range of ages and two delays (5 min and 24 hr.) to characterise any age-dependent changes in object novelty and/or spatial novelty detection. Mice were initially presented with either 2 identical or four different objects for three separate sample phases prior to the test phase. Supplementary Table 1 shows the mean contact time with 2 identical and 4 different objects across 3 sample phases of PDAPP mice and WT littermate controls at 6-8, 10-12 and 14-16 months of age. Results of a repeated measures ANOVA of contact times with 2 identical objects revealed a decline in contact time with objects across sample phases *F*_(1.6, 43.6)_ = 271.8, p<0.001 and overall WT mice explored objects more than PDAPP mice, *F*_(1, 27)_ = 8.6, p<0.01. A significant genotype x sample phase interaction *F*_(1.6, 43.6)_ = 16.8, p<0.001 further revealed that WT mice explored objects more than PDAPP mice in sample phase 1, *F*_(1, 27)_ = 21.3, p<0.001, and sample phase 2 *F*_(1, 27)_ = 5.8, p<0.05, but not at sample phase 3, *F*_(1, 27)_ = 1.9, p>0.05. Despite this difference in contact times between genotypes, both PDAPP, *F*_(2, 26)_ = 56.2, p<0.001, and WT mice, *F*_(2, 26)_ = 161.8, p<0.001 showed a significant reduction in contact times with objects in sample phase 3 compared to sample phase 1, indicating short-term habituation to the 2 identical objects in both PDAPP and WT littermates.

Similar results were reported in contact times with 4 different objects. ANOVA results showed a significant reduction in contact times across sample phases, *F*_(2, 54)_ = 277.8, and WT mice explored objects more than PDAPP mice overall, *F*_(1, 27)_ = 22.9, p<0.001, p<0.001. A significant genotype x age interaction was also reported, *F*_(2, 54)_ = 3.8; WT mice explored objects more than PDAPP mice at 10-12 months of age, *F*_(1, 27)_ = 16.0, p<0.001 and at 14-16 months of age *F*_(1, 27)_ = 14.5, p<0.001, but not at 6-8 months of age, *F*_(1, 27)_ = 3.3, p>0.05. At 14-16 months of age, WT mice showed an increase in contact time compared to their younger age points, *F*_(1, 27)_ = 6.0, p<0.01. This was not observed in PDAPP mice, which explored objects at similar level at all ages, *F*_(2, 26)_ = 0.2, p>0.05. Finally, a significant genotype x sample phase interaction, *F*(2, 54) = 19.9, p<0.001 revealed that WT mice explored objects more that PDAPP mice at sample phase 1, *F*_(1, 27)_ = 56.3, p<0.001, sample phase 2, *F*_(1, 27)_ = 14.6, p<0.01, and sample phase 3, *F*_(1, 27)_ = 8.0, p<0.05. Despite this, both PDAPP, *F*_(2, 26)_ = 57.6, p<0.001 and WT mice *F*_(2, 26)_ = 189.5, p<0.001 showed a reduction in contact times in sample phase 3 compared to sample phase 1. This indicated that both groups of mice showed short-term habituation to the 4 different objects across the sample phases.

**Supplementary Table 2. PDAPP mice showed an age-dependent decline in object-in-place memory but not object novelty detection.**

| Age | Genotype | Mean contact time during the sample phases (seconds) with all objects | | | |
| --- | --- | --- | --- | --- | --- |
|  | | Novel Stimulus  (5 min) | Familiar Stimulus  (5 min) | Novel Stimulus (24 hr) | Familiar Stimulus  (24 hr) |
|  |  | Object-Novelty Task (2 Identical Objects) | | | |
| 6-8 Months | WT | 18.97 | 5.37 | 20.26 | 10.57 |
|  | PDAPP | 10.79 | 3.55 | 10.62 | 7.33 |
| 10-12 Months | WT | 19.52 | 6.55 | 19.27 | 12.80 |
|  | PDAPP | 11.39 | 4.10 | 7.03 | 5.09 |
| 14-16 Months | WT | 33.81 | 8.13 | 21.29 | 10.55 |
|  | PDAPP | 18.01 | 5.52 | 11.41 | 6.45 |
|  | | Object-in-Place Task (4 Different Objects) | | | |
| 6-8 Months | WT | 18.93 | 8.59 | 16.64 | 11.92 |
|  | PDAPP | 9.69 | 5.02 | 11.81 | 9.56 |
| 10-12 Months | WT | 20.44 | 10.08 | 26.82 | 16.36 |
|  | PDAPP | 10.25 | 6.20 | 10.71 | 7.37 |
| 14-16 Months | WT | 24.57 | 11.08 | 27.52 | 15.92 |
|  | PDAPP | 8.81 | 6.69 | 9.82 | 8.42 |

Supplementary Table 2: Mean contact times (seconds) with objects in the test phases for both object novelty and object-in-place tasks. Contact times are displayed for each age and delay tested.

Supplementary Table 2 shows the mean contact times with objects during the object-novelty and OiP test phase for PDAPP mice and WT littermate controls. A 4-way ANOVA of the contact times during the object-novelty test phase revealed that WT mice explored the objects more than PDAPP mice, *F*_(1, 27)_ = 22.1, p<0.001, whilst overall the mice still explored novel objects more than familiar objects (collapsed across genotype), *F*_(1, 27)_ = 351.1, p<0.001. A significant object x genotype interaction, *F*_(1, 27)_ = 18.0, p<0.001 showed that WT mice explored both the novel object, *F*_(1, 27)_ = 22.5, p<0.001 and familiar object, *F*_(1, 27)_ = 19.7, p<0.001, more than PDAPP mice. However, both the WT mice, *F*_(1, 27)_ = 273.6, p<0.001 and PDAPP mice, *F*_(1, 27)_ = 101.5, p<0.001 explored the novel object in preference to the familiar object in the test phase. There were no significant interactions involving transgenic group and delay.

Analysis of contact times with objects in the OiP test phase revealed similar results to the object-novelty test. WT mice again showed a greater contact time with objects compared to PDAPP mice, *F*_(1, 27)_ = 25.8, p<0.001, although mice overall showed a preference for objects in novel spatial arrangements relative to those in a familiar location, *F*_(1, 27)_ = 276.6. A significant age x place x genotype interaction was reported, *F*_(2, 54)_ = 4.6, p<0.05. This interaction further showed that WT mice explored both novel and familiar object arrangements more than PDAPP mice across all ages (minimal value = *F*_(1, 27)_ = 8.3, p<0.01, familiar exploration at 14-16 months of age). Despite this, both WT and PDAPP mice still showed a preference to explore objects in novel locations more than familiar locations at 6-8 and 10-12 months of age. However, only WT mice showed this preference at 14 -16 months of age (minimal effect; PDAPP mice at 10-12 months of age, *F*_(1, 27)_ = 23.6, p<0.001). PDAPP mice at 14-16 months of age showed no preference to objects in novel locations, *F*_(1, 27)_ = 3.2, p>0.05. This result showed an age-dependent impairment in the ability of PDAPP mice to detect object-location mismatches. The fat that object novelty detection was not affected at the same age suggest the deficit in OiP memory was not a product of gross sensory-motor changes in the old PDAPP mice.

**Supplementary Table 3 & 4. 2B3 administration caused no change in object contact times.**

| Treatment Group | Before/After Treatment | Mean contact time during the sample phases with all objects (s) | | |
| --- | --- | --- | --- | --- |
|  | | Sample Phase 1 | Sample Phase 2 | Sample Phase 3 |
| WT Untreated | Pre | 43.60 | 28.84 | 23.40 |
|  | Post | 43.52 | 24.32 | 17.56 |
| WT Vehicle | Pre | 34.53 | 25.17 | 19.47 |
|  | Post | 27.16 | 15.67 | 16.35 |
| PDAPP Vehicle | Pre | 17.10 | 9.95 | 8.22 |
|  | Post | 17.60 | 9.06 | 4.80 |
| PDAPP 2B3 | Pre | 24.23 | 15.95 | 12.46 |
|  | Post | 23.10 | 15.71 | 10.30 |

Supplementary Table 3A: Mean contact time data (seconds) with objects in the sample phase for the object-in-place task. Mean contact times are shown for mice before (Pre) and following (Post) ICV 2B3 administration.

Analysis of the sample phase data following 2B3 administration (Supplementary Table 3) was carried out by a repeated measures ANOVA. This analysis showed that all mice reduced contact times with objects across sample phases, *F*_(1.6, 63.2)_ = 88.4, p<0.001. However, there was a significant difference in total contact time between treatment groups, *F*_(1, 39)_ = 16.9, p<0.001. A post-hoc Tukey analysis revealed that WT mice explored objects significantly more than PDAPP Vehicle mice, p<0.001 and PDAPP 2B3 mice, p<0.01. No significant difference in contact times was reported between the PDAPP treatment groups, p>0.1. These results indicate that 2B3 administration did not alter the lower object contact times or normal habituation of object exploration in PDAPP mice.

| Treatment Group | Before/After Treatment | Mean contact time during the sample phases with all objects (s) | | |
| --- | --- | --- | --- | --- |
| Novel Object Task (2 Items) | | | | |
|  | | Sample Phase 1 | Sample Phase 2 | Sample Phase 3 |
| WT Untreated | Pre | 21.15 | 19.12 | 14.21 |
|  | Post | 25.31 | 21.23 | 17.68 |
| WT Vehicle | Pre | 19.25 | 14.51 | 9.78 |
|  | Post | 22.93 | 17.78 | 10.35 |
| PDAPP Vehicle | Pre | 20.15 | 14.22 | 9.58 |
|  | Post | 15.54 | 14.98 | 11.56 |
| PDAPP 2B3 | Pre | 17.48 | 14.97 | 10.06 |
|  | Post | 13.96 | 9.51 | 6.98 |
| Object in Place Task | | | | |
|  | | Sample Phase 1 | Sample Phase 2 | Sample Phase 3 |
| WT Untreated | Pre | 35.74 | 23.71 | 17.04 |
|  | Post | 43.92 | 38.23 | 24.97 |
| WT Vehicle | Pre | 28.96 | 18.54 | 14.44 |
|  | Post | 38.11 | 30.50 | 21.37 |
| PDAPP Vehicle | Pre | 27.69 | 22.93 | 15.69 |
|  | Post | 27.00 | 20.76 | 13.63 |
| PDAPP 2B3 | Pre | 30.32 | 21.56 | 16.68 |
|  | Post | 26.40 | 19.37 | 17.57 |

Supplementary Table 3B: Mean contact time data (seconds) with objects in the sample phase for the novel object and object-in-place task. Mean contact times are shown for mice before (Pre) and following (Post) peripheral 2B3 administration.

Analysis of the sample phase data following peripheral 2B3 administration (Supplementary Table 3b) was carried out by a repeated measures ANOVA. The analysis of sample phase contact time data of the novel object task showed that all mice reduced in contact times with objects across sample phases, *F*_(1.5, 50.4)_ = 88.4, p<0.001. However, there was a significant difference in total contact time between treatment groups, *F*_(2, 34)_ = 3.8, p<0.05. A post-hoc Tukey analysis revealed that WT mice explored objects significantly more than PDAPP 2B3 mice, p<0.05, but not PDAPP vehicle mice, p>0.1. No significant difference in contact times were reported between the PDAPP treatment groups, p>0.1. Sample phase contact time analysis for the OiP task revealed a significant reduction in contact time with objects across sample phases, *F*_(2, 68)_ = 64.4, p<0.001. However, there was no significant difference in total contact time between treatment groups as reported in the novel object task, *F*_(2, 34)_ = 1.1, p>0.1. Collectively, these results indicate that 2B3 administration did not alter the lower object contact times with two identical objects or normal habituation of object exploration in PDAPP mice in both recognition memory tasks.

| Treatment Group | Before/After Treatment | Mean contact time during the sample phases with all objects (s) | | |
| --- | --- | --- | --- | --- |
| Novel Object Task | | | | |
|  | | Sample Phase 1 | Sample Phase 2 | Sample Phase 3 |
| WT Control | Pre | 42.68 | 43.30 | 43.50 |
|  | Post | 50.84 | 42.17 | 31.17 |
| WT 2B3 | Pre | 63.45 | 44.81 | 43.17 |
|  | Post | 43.89 | 34.01 | 29.23 |
| Object in Place Task | | | | |
|  | | Sample Phase 1 | Sample Phase 2 | Sample Phase 3 |
| WT Control | Pre | 72.57 | 93.68 | 71.36 |
|  | Post | 69.13 | 71.60 | 55.83 |
| WT 2B3 | Pre | 105.26 | 103.80 | 71.36 |
|  | Post | 90.81 | 80.37 | 65.26 |

Supplementary Table 3C: Mean contact time data (seconds) with objects in the sample phase for the object novelty task object-in-place task for WT mice administered 2B3 or IgG1κ control mice. Mean contact times are shown for WT mice before (Pre) and following (Post) ICV 2B3 administration.

Sample phase contact time data with 2 identical objects showed no main effect of group, *F*(1, 13)=0.01, p>0.5, a significant main effect of time point (pre/post), *F*(1, 13)=6.25, p<0.05, but no main group x time point interaction, *F*(1, 13)=3.87, p>0.05. A main effect of sample phase was further reported, *F*(2, 26)=4.60, p<0.05, but not sample phase x group interaction, *F*(2, 23)=0.71, p>0.5. No further main effects or interactions (sample phase x time point interaction, *F*(2, 26)=0.53, p>0.5, and group x sample phase x time point interaction, *F*(2, 23)=1.33, p>0.1) were reported. A main effect of sample phase revealed that although mice showed a pattern to explore objects less by sample phase 3 compared to sample phase 1, this was not significant (p=0.066). Collectively these data suggest that 2B3 administration did not effect overall object exploration of 2 identical objects across sample trials.

Analysis of contact times of four different objects (in OiP sample phases) revealed a main effect of sample phase, *F*(2, 26)=8.80, p<0.001, no significant sample phase x group interaction, *F*(2, 26)=2.23, p>0.1, no significant main effect of time point (pre/post), *F*(1, 13)=3.22, p>0.05, no time point x group interaction, *F*(1, 13)=0.02, p>0.5, no significant sample phase x time point interaction *F*(2, 26)=0.94, p>0.1 and no sample phase x time point x group *F*(2, 26)=0.32, p>0.5. Pairwise comparisons for a main effect of sample phase revealed mice explored objects in sample phase 3 significantly less than in sample phase 1 (p<0.05) and sample phase 2 (p<0.01). No difference was reported between sample phase 1 and sample phase 2 (p>0.5). Collectively, these data suggest that mice habituated to objects across sample phases, however this effect was unchanged by 2B3 administration.

| Treatment Group | Pre-/Post-Treatment | Mean contact time during the test phase with objects in novel and familiar spatial locations (s) | |
| --- | --- | --- | --- |
|  | | Novel Object Location | Familiar Object Location |
| WT Untreated | Pre | 30.51 | 13.51 |
|  | Post | 23.82 | 11.40 |
| WT Vehicle | Pre | 16.01 | 8.77 |
|  | Post | 18.32 | 9.98 |
| PDAPP Vehicle | Pre | 6.51 | 4.85 |
|  | Post | 6.53 | 4.87 |
| PDAPP 2B3 | Pre | 12.78 | 9.02 |
|  | Post | 13.19 | 7.14 |

Supplementary Table 4A: Mean contact time data (seconds) with objects in the test phase for the object-in-place task. Mean contact times are shown for mice before (Pre) and following (Post) ICV 2B3 administration.

Supplementary Table 4 shows the mean contact times with objects in novel and familiar locations in the OiP test phase. A 3-way repeat measures ANOVA revealed different contact times between treatment groups, *F*_(2, 39)_ = 10.1, p<0.001. Post-hoc Tukey analysis determined that WT mice had higher contact times with objects than either PDAPP Vehicle mice, p<0.01 and PDAPP 2B3 treated mice, p<0.05. No significant difference in contact times were observed between PDAPP treatment group, p>0.1. Overall mice showed a preference to explore objects in novel locations over familiar, *F*_(1, 39)_ = 84.1, p<0.001. However a significant object location x treatment group interaction, *F*(2, 39) = 15.5, p<0.001 showed that all treatment groups had an ability to discriminate objects in novel locations when data were collapsed across pre- and post-treatment times, all p’s<0.05. However, WT mice explored objects in novel locations more than PDAPP Vehicle mice, p<0.001, and PDAPP 2B3 mice, p<0.01. WT mice further explored objects in familiar locations more than PDAPP V mice, p<0.01, but not PDAPP 2B3 mice, p>0.1. No significant difference was reported between PDAPP mice for objects in novel or familiar locations, both p’s>0.1. The object location x time x treatment group interaction failed to reach significance, *F*_(2, 39)_ = 2.8, p>0.05.

| Treatment Group | Pre-/Post-Treatment | Mean contact time during the test phase with objects in novel and familiar spatial locations (s) | |
| --- | --- | --- | --- |
| Novel Object Task | | | |
|  | | Novel Object | Familiar Object |
| WT Untreated | Pre | 19.15 | 6.41 |
|  | Post | 23.92 | 6.15 |
| WT Vehicle | Pre | 12.09 | 5.59 |
|  | Post | 20.49 | 6.44 |
| PDAPP Vehicle | Pre | 12.72 | 4.66 |
|  | Post | 13.05 | 3.59 |
| PDAPP 2B3 | Pre | 13.72 | 3.94 |
|  | Post | 14.43 | 4.25 |
| Object-in-Place Task | | | |
|  | | Novel Object Location | Familiar Object Location |
| WT Untreated | Pre | 18.06 | 9.30 |
|  | Post | 24.57 | 14.16 |
| WT Vehicle | Pre | 14.73 | 6.32 |
|  | Post | 18.18 | 8.69 |
| PDAPP Vehicle | Pre | 12.28 | 6.70 |
|  | Post | 9.89 | 6.83 |
| PDAPP 2B3 | Pre | 13.59 | 7.49 |
|  | Post | 14.75 | 7.27 |

Supplementary Table 4B: Mean contact time data (seconds) with objects in the test phase for the novel object and object-in-place task. Mean contact times are shown for mice before (Pre) and following (Post) peripheral 2B3 administration.

Supplementary Table 4b shows the mean contact times with novel and familiar objects in the novel object test phase and objects in novel and familiar locations in the OiP test phase. A 3-way repeat measures ANOVA revealed that mice showed different contact times between treatment groups in the novel object task, *F*_(2, 34)_ = 3.5, p<0.05, but not the OiP task, *F*_(2, 34)_ = 1.6, p>0.1 when data were collapsed across pre- and post-treatment times. Post-hoc Tukey analysis determined, however, that there were no significant differences between any treatment groups in the novel object task (maximal effect WT vs PDAPP vehicle, p=0.063). In both tasks animals showed a preference to explore novel objects, *F*_(1, 34)_ =101.4, p<0.001, and objects in novel locations, *F*_(1, 34)_ = 106.8, p<0.001 over familiar when data were collapsed across pre- and post-treatment times. Neither the novel object task nor the OiP task showed a significant time * object(location) * group interaction, *F*_(2 34)_ = 1.7, p=0.19, *F*_(2 34)_ = 1.6, p=0.21 respectively. No further main effects or interactions were reported.

| Treatment Group | Pre-/Post-Treatment | Mean contact time during the test phase with objects in novel and familiar spatial locations (s) | |
| --- | --- | --- | --- |
| Novel Object Task | | | |
|  | | Novel Object | Familiar Object |
| WT Control | Pre | 46.48 | 11.12 |
|  | Post | 41.45 | 11.33 |
| WT 2B3 | Pre | 45.23 | 13.36 |
|  | Post | 48.86 | 12.04 |
| Object-in-Place Task | | | |
|  | | Novel Object Location | Familiar Object Location |
| WT Control | Pre | 52.87 | 27.84 |
|  | Post | 50.88 | 29.59 |
| WT 2B3 | Pre | 60.29 | 30.07 |
|  | Post | 30.59 | 32.97 |

Supplementary Table 4C: Mean contact time data (seconds) with objects in the test phase for the object-in-place task for WT mice administered 2B3 or IgG1κ control mice. Mean contact times are shown for mice before (Pre) and following (Post) ICV 2B3 administration.

Analysis of object novelty test phase data revealed no significant main effect of group, *F*(1, 13)=0.29, no main effect of time point, *F*(1, 13)=0.03, p>0.5 and no time point x group interaction, *F*(1, 13)=0.24, p>0.5. A significant main effect of object, *F*(1, 13)=52.60, p<0.001, was reported, but no object x group interaction, *F*(1, 13)=0.03, p>0.05. No further main effects or interactions (object x time point, *F*(1, 13)=0.001, p>0.5, and object x time point x group interaction, *F*(1, 13)=0.72, p>0.1) were reported. Simple main effects analysis of a main effect of object revealed that mice explored the novel object significantly more than the familiar. Collectively these data indicate that mice were able to discriminate novel objects. This effect was unaltered by 2B3 administration.

Further analysis of the contact times with objects in novel and familiar locations during the test phase revealed no significant main effect of group, *F*(1, 13)=0.14, p>0.5, no significant main effect of time point (pre/post), *F*(1, 13)=3.26, p>0.05 or time point x group interaction, *F*(1, 13)=3.16, p>0.05. A significant main effect of location was reported, *F*(1, 13)=43.99, p<0.0001, although no significant location x group interaction was reported, *F*(1, 13)=2.73, p>0.1. No further significant interactions (time point x location, *F*(1, 13)=4.43, p>0.05 and time point x location x group, *F*(1, 13)=2.79, p>0.1) were reported. Pairwise comparisons of the main effect of location revealed that when data were collapsed across group and time-point, mice explored objects in novel locations for a greater time than objects in familiar locations. Collectively these data show that, although numerically, following 2B3 administration WT mice showed reduced contact times with objects in novel locations, this was not confirmed statistically (no significant time point x location x group interaction). However, mice otherwise showed a preference to explore objects in novel locations over familiar.

**Supplementary Table 5. Peripheral administration of 2B3 showed no change in foraging behaviours in PDAPP mice.**

| Group | Drug Time-Point | Time Taken (s) |
| --- | --- | --- |
| WT Vehicle | Pre | 235.28 |
|  | Post | 173.53 |
| PDAPP Vehicle | Pre | 173.30 |
|  | Post | 185.98 |
| PDAPP 2B3 | Pre | 169.89 |
|  | Post | 170.50 |

Supplementary Table 5: Mean foraging time for mice before (Pre) and following (Post) peripheral 2B3 administration.

Total Time

Total time taken was analysed by 3x2 ANOVA. A significant time x group interaction was reported, *F*(_2, 34_) = 5.2, p<0.01. Tests for simple main effects further revealed that WT mice took less time to complete the task at the post-treatment time compared to their pre-treatment scores. PDAPP vehicle and PDAPP 2B3 mice showed no change in times taken to complete the task when comparing pre- and post-treatment times. No further effects were reported.

**Supplementary Tables 6 & 7. The NR2B receptor antagonist Ro25-6981 altered contact times with objects in the OiP test but not during sample exposure.**

| Genotype | Treatment | Mean contact time during the sample phases with all objects (s) | | |
| --- | --- | --- | --- | --- |
|  | | Sample Phase 1 | Sample Phase 2 | Sample Phase 3 |
| WT | Vehicle | 29.33 | 24.30 | 22.69 |
|  | Ro25-6981 | 27.64 | 20.54 | 17.09 |

Supplementary Table 6: Mean contact time data (seconds) with objects in the sample phase for the object-in-place task. Mean contact times are shown for mice following vehicle and Ro25-6981 administration.

Supplementary Table 5 shows the mean contact time with 4 different objects across 3 sample phases of WT mice receiving vehicle and Ro25-6981 at 17-18 months of age. Results of a repeated measures ANOVA revealed that all mice showed a decline in contact times with objects across sample phases, *F*_(1.5, 27.6_) = 12.7. No further main effects or interactions were observed.

| Genotype | Treatment | Mean contact time during the test phase with objects in novel and familiar spatial locations (s) | |
| --- | --- | --- | --- |
|  | | Novel Object Location | Familiar Object Location |
| WT | Vehicle | 16.83 | 9.96 |
|  | Ro25-6981 | 12.31 | 9.25 |

Supplementary Table 7: Mean contact time data (seconds) with objects in the test phase for the object-in-place task. Mean contact times are shown for mice following vehicle and Ro25-6981 administration.

Analysis of contact times with objects in the test phase (Supplementary Table 7) revealed a significant main effect of object location, *F*(_1, 18_) = 72.7, p<0.001, a significant object location x group interaction, *F*(_1, 18_) = 10.8, p<0.01, but no significant main effect of group, *F*(_1, 18_) = 0.5, p>0.5. Test for simple main effects revealed that both vehicle (p<0.001) and Ro25 (p<0.01) administered mice explored objects in novel locations in preference to familiar. Although numerically Ro25 administered mice explored objects in novel locations less than vehicle administered mice, thiere was no significant difference in exploration of objects in novel locations p>0.1, or familiar locations, p>0.5. Collectively these data show that Ro25-6981 administration did not affect overall object exploration and although mice appeared to explore novel object arrangements less, still maintained a level of discrimination

**Supplementary Figure 1. ICV 2B3 administration to WT mice causes associative recognition memory impairment and reduced endogenous Aβ40**

Supplementary Figure 1. **2B3 administration in WT mice caused impaired OiP memory performance and reduced endogenous Aβ40 without affecting ON memory and endogenous levels of APP.** (A) All mice at both pre- and post-administration stages were able to perform the object-novelty task significantly above chance, ***p<0.001, **p<0.01. (B) WT mice administered ICV 2B3 showed an impaired OiP memory performance compared to chance (0.5), p>0.5 whilst IgG1κ control administered WT mice were significantly above chance, **p<0.01. (C-D) No overall changes in total levels of soluble APP or BACE1 were detected in hippocampal homogenates, as determined by Western blot. (E) 2B3 administration caused a significant reduction in endogenous soluble Aβ40 compared to IgG1κ control WT mice, *p<0.05. A-B data were analysed using mixed measures ANOVA and individual DR scores compared to chance using one-sample t-test. APP, BACE1 and soluble Aβ40 levels were compared by independent samples t-test. Error bars represent the standard error of the mean (SEM).

Following 2B3 administration, WT mice maintained the ability to discriminate novel objects (Supplementary Figure 1A) and were comparable to control IgG1κ administered mice (no main effect of group, *F*(1, 13)=0.79, p>0.1 or group x time point interaction, *F*(1, 13)=0.78, p>0.1). Moreover all DR scores were significantly above chance (minimal effect: 2B3 post-treatment, t(6)=5.41, p<0.01). However, OiP DR scores (Supplementary Figure 1B) showed a decline in OiP performance following 2B3 administration. 2x2 ANOVA analysis revealed a significant main effect of group, *F*(1, 13)=8.76, p<0.05, but no group x time point interaction, *F*(1, 13)=2.86, p>0.1. When analysing DR scores against chance (0.5), one-sample t-tests revealed that control pre- and post-treatment and 2B3 pre-treatment were significantly above chance (minimal effect IgG1κ control post-treatment, t(7)=3.58, p<0.01). However, following 2B3 treatment, mice did not perform significantly above chance, t(6)=0.49, p>0.5. These data indicate that 2B3 impaired OiP performance, however ON performance remained intact following 2B3 administration.

To determine whether 2B3 administration altered endogenous levels of APP or BACE1, hippocampal homogenates were analysed by Western blot (Supplementary Figure 1C-D). No significant changes in the levels of BACE1, t(13)=0.5, p>0.5 or APP t(13)=0.81, p>0.5 were reported. To further confirm inhibition of APP processing by 2B3, total levels of endogenous soluble Aβ were analysed by ELISA. Endogenous soluble Aβ42 levels were undetectable by ELISA, however, soluble Aβ40 (Supplementary Figure 1E) levels were significantly reduced in mice administered 2B3 compared to IgG1κ antibody control mice, t(13)=2.45, p<0.05.

**Supplementary Analysis 1A. Treatment groups were balanced across contact times and DR scores for 2B3 ICV administration**

Prior to minipump implantation, WT and PDAPP mice were divided into 4 groups; WT untreated (WT UT), WT vehicle (WT V), PDAPP vehicle (PDAPP V) and PDAPP 2B3. To ensure that the groups were matched in terms of their surgical assignments two separate 2x2 repeat measures ANOVAs were carried out on sample phase and test phase contact time data and a one-way ANOVA was performed with post-hoc Tukey analysis on DR data shown in Supplementary Tables 2, 3 and Fig 1B.

Contact time data, violated Levene’s test of equality of error variances, p<0.05. Therefore, the data were transformed by square root transform. Although transformation resulted in the data no longer violating this test, Mauchly’s test of Sphericity remained violated, p<0.05. Therefore, Greenhouse-Geiser comparisons were reported. Repeated measures ANOVA revealed a significant main effect of sample phase, *F*(1.7, 63.5) = 50.2, p<0.001, no significant sample phase x treatment group interaction, *F*(5.0, 63.5) = 0.6, p>0.5 and a significant main effect of treatment group, *F*(3, 38) = 5.6, p<0.01. Post-hoc Tukey analysis of the significant main effect of treatment group revealed that only WT UT mice explored objects (when collapsed across sample phase) more than PDAPP V mice, p<0.01. No further significant differences in total contact times were reported (WT V vs PDAPP V, p=0.052).

A repeat measures ANOVA analysed the pre-treatment contact times in the aged study test phase. The contact times data summarised in Table 3 were transformed by square root due to violations in Levene’s Test of Equality of Error Variance. The analysis reported a significant main effect of object location, *F*(1, 38) = 70.4, p<0.001, a significant object location x treatment group interaction, *F*(3, 38) = 10.3, p<0.001 and a significant main effect of treatment group, *F*(3, 38) = 9.1, p<0.001. Post-hoc Tukey analysis of the between-subject main effect collapsed across object location revealed no significant difference between WT UT and WT V mice, p>0.1. WT UT mice showed a greater contact time with objects than PDAPP V, p<0.001 and PDAPP 2B3, p<0.01. No further significant differences between treatment group were reported. Because there was a significant object location x treatment group interaction, tests for simple main effects were performed. Between-subjects comparisons revealed that WT UT mice explored objects in novel locations more than both PDAPP groups, p<0.001. WT V mice explored objects in novel locations more than PDAPP V mice only. No further significant effects were reported when the novel location data. WT UT mice showed a greater contact time with objects in familiar locations than PDAPP V mice only, p<0.05. No further differences in contact times with objects in familiar locations were reported. Within-subjects analysis to determine if objects in novel locations were explored significantly more than familiar locations revealed that both WT UT and WT V mice explored objects in novel locations significantly more than objects in familiar locations, p<0.001. Neither PDAPP V or PDAPP 2B3 mice explored objects in novel locations more than objects in familiar locations, both p’s >0.05.

One-way ANOVA analysis of DR scores revealed a significant main effect of group, *F*(3, 41) = 5.8, p<0.01. Post-hoc analysis revealed no significant differences between either WT group, p>0.5, or PDAPP group, p>0.5. The only significant differences were reported when comparing WT groups to PDAPP groups respectively, p’s <0.05. Taken together, this pre-treatment analysis showed that mice habituated to objects across sample phases without differential effects across treatment group as determined by a lack of sample phase x treatment group interaction. Test phase contact times revealed that both WT groups showed a discrimination toward objects in novel locations over objects in familiar locations, where as both treatment groups of PDAPP mice did not. This was further observed when contact times were converted to DRs, both WT groups showed significantly greater DR scores than either PDAPP group.

**Supplementary Analysis 1B. Treatment groups were balanced across contact times and DR scores for peripheral 2B3 administration**

Prior to intraperitoneal (IP) administration of 2B3, WT and PDAPP mice were divided into 4 groups; WT untreated (WT UT), WT vehicle (WT V), PDAPP vehicle (PDAPP V) and PDAPP 2B3. To ensure that the groups were matched in terms of their treatment assignments separate repeat measures ANOVAs were carried out on sample phase and test phase contact time data and a one-way ANOVA was performed with post-hoc Tukey analysis on DR data shown in Supplementary Tables 3B, 4B and Fig 2B.

Sample phase contact time (supplementary table 3B) data analysis revealed a significant main effect of sample phase, *F*(2, 66) = 53.9, p<0.001, no significant sample phase x treatment group interaction, *F*(6, 66) = 0.8, p>0.5 and no significant main effect of treatment group, *F*(3, 33) = 0.3, p>0.5.

A repeat measures ANOVA analysed the pre-treatment contact times in the test phase of the OiP task. The contact times data summarised in supplementary table 4B analysis reported a significant main effect of object location, *F*(1, 33) = 33.5, p<0.001, a non significant object location x treatment group interaction, *F*(3, 33) = 0.4, p>0.5 and a non significant main effect of treatment group, *F*(3, 33) = 0.4, p>0.5.

One-way ANOVA analysis of DR scores revealed no significant main effect of group, *F*(3, 33) = 0.2, p>0.5.

Collectively, these analyses show there was no significant differences in object contact times or DR scores prior to the onset of treatment.

**Supplementary Analysis 2A. Justification of collapsing across WT untreated and vehicle administered mice**

Sample phase and test phase contact times and DR score data were compared by mixed measures ANOVA using time (pre- and post-treatment) and group as factors across all analysis and sample phase and location as further factors respectively.

Sample phase contact analysis by mixed measures ANOVA revealed no significant main effect of time (pre-treatment and post-treatment), *F*_(1, 19)_ = 0.9, p>0.1, no significant main effect of treatment group, *F*_(1, 19)_ = 2.9, p>0.1 and no significant time x treatment group interaction, *F*_(1, 19)_ = 0.04, p>0.5. Contact times with objects in the test phase revealed no significant main effect of time, *F*_(1, 19)_ = 0.7, p>0.1, no significant main effect of treatment group, *F*_(1, 19)_ = 2.5, p>0.1 and no significant time x treatment group interaction, *F*_(1, 19)_ = 1.7, p>0.1. Finally, DR scores were analysed using a 2x2 repeated measures ANOVA. No significant main effect of time, *F*_(1, 19)_ = 0.8 p>0.5, treatment group, *F*_(1, 19)_ = 0.13, p>0.5 and no significant treatment group x treatment time interaction, *F*_(1, 19)_ = 0.1, p>0.5 was reported. Therefore, no difference between either WT group was observed across all contact time measures and DR scores. This implies that neither osmotic mini-pump insertion or vehicle administration had any gross impact on WT behaviour. For this reason WT groups were collapsed for all subsequent analyses. Analysis of 2B3 intervention was thus carried out with 3 main treatment groups; WT control, PDAPP vehicle administered mice and 2B3 administered PDAPP mice.

**Supplementary Analysis 2B. Justification of collapsing across WT untreated and vehicle administered mice**

Sample phase and test phase contact times and DR score data were compared by mixed measures ANOVA using time (pre- and post-treatment) and group as factors across all analysis and sample phase and location as further factors respectively.

Sample phase contact analysis by mixed measures ANOVA revealed no significant main effect of treatment group, *F*(1, 16) = 0.7, p>0.1, a significant main effect of sample phase, *F*(2, 32) = 40.6, p<0.001, a non-significant sample phase x treatment group interaction, *F*(2, 32) = 0.5, p>0.5, a significant main effect of time (pre-treatment and post-treatment), *F*(1, 16) = 12.6, p<0.01, and no significant time x treatment group interaction, *F*(1, 16) = 0.03, p>0.5.

Contact times with objects in the test phase revealed no significant main effect of treatment group, *F*(1, 16) = 1.3, p>0.1, no significant main effect of time, *F*(1, 16) = 3.2, p>0.05, no significant time x treatment group interaction, *F*(1, 16) = 0.3, p>0.5, a significant main effect of location, *F*(1, 16) = 27.2, p<0.001, a non-significant location x treatment group interaction, *F*(1, 16) = 0.01, p>0.5.

DR scores were analysed using a 2x2 repeated measures ANOVA. Analysis reported no significant main effect of treatment group, *F*(1, 16) = 0.4, p>0.5, no significant main effect of time, *F*(1, 16) = 0.1 p>0.5, and no significant treatment group x treatment time interaction, *F*(1, 16) = 0.1, p>0.5 was reported. Therefore, no difference between either WT group was observed across all contact time measures and DR scores. This implies that long-term IP injections and vehicle administration had no gross impact on WT behaviour. For this reason WT groups were collapsed for all subsequent analyses. Analysis of 2B3 intervention was thus carried out with 3 main treatment groups; WT control, PDAPP vehicle administered mice and 2B3 administered PDAPP mice.
